# A nomogram model for predicting 5-year risk of prediabetes in Chinese adults

**DOI:** 10.1038/s41598-023-50122-3

**Published:** 2023-12-18

**Authors:** Yanhua Hu, Yong Han, Yufei Liu, Yanan Cui, Zhiping Ni, Ling Wei, Changchun Cao, Haofei Hu, Yongcheng He

**Affiliations:** 1 College of Information Science and Engineering, Liuzhou Institute of Technology, Liuzhou, 545616 Guangxi Zhuang Autonomous Region China; 2 Department of Emergency, Shenzhen Second People’s Hospital, Shenzhen, 518000 Guangdong Province China; 3 Department of Emergency, The First Affiliated Hospital of Shenzhen University, Shenzhen, 518000 Guangdong Province China; 4 Department of Neurosurgery, Shenzhen Second People’s Hospital, Shenzhen, 518000 Guangdong Province China; 5 Department of Neurosurgery, The First Affiliated Hospital of Shenzhen University, Shenzhen, 518000 Guangdong Province China; 6 Department of Rehabilitation, Shenzhen Dapeng New District Nan’ao People’s Hospital, No. 6, Renmin Road, Dapeng New District, Shenzhen, 518000 Guangdong Province China; 7 Department of Nephrology, Shenzhen Second People’s Hospital, No. 3002 Sungang Road, Futian District, Shenzhen, 518000 Guangdong Province China; 8 Department of Nephrology, The First Affiliated Hospital of Shenzhen University, Shenzhen, 518000 Guangdong Province China; 9 Department of Nephrology, Shenzhen Hengsheng Hospital, No. 20 Yintian Road, Baoan District, Shenzhen, 518000 Guangdong Province China; 10 Department of Nephrology, Affiliated Hospital of North Sichuan Medical College, Nanchong, 637000 Sichuan China

**Keywords:** Diseases, Endocrinology, Health care

## Abstract

Early identification is crucial to effectively intervene in individuals at high risk of developing pre-diabetes. This study aimed to create a personalized nomogram to determine the 5-year risk of pre-diabetes among Chinese adults. This retrospective cohort study included 184,188 participants without prediabetes at baseline. Training cohorts (92,177) and validation cohorts (92,011) were randomly assigned (92,011). We compared five prediction models on the training cohorts: full cox proportional hazards model, stepwise cox proportional hazards model, multivariable fractional polynomials (MFP), machine learning, and least absolute shrinkage and selection operator (LASSO) models. At the same time, we validated the above five models on the validation set. And we chose the LASSO model as the final risk prediction model for prediabetes. We presented the model with a nomogram. The model's performance was evaluated in terms of its discriminative ability, clinical utility, and calibration using the area under the receiver operating characteristic (ROC) curve, decision curve analysis, and calibration analysis on the training cohorts. Simultaneously, we also evaluated the above nomogram on the validation set. The 5-year incidence of prediabetes was 10.70% and 10.69% in the training and validation cohort, respectively. We developed a simple nomogram that predicted the risk of prediabetes by using the parameters of age, body mass index (BMI), fasting plasma glucose (FBG), triglycerides (TG), systolic blood pressure (SBP), and serum creatinine (Scr). The nomogram's area under the receiver operating characteristic curve (AUC) was 0.7341 (95% CI 0.7290–0.7392) for the training cohort and 0.7336 (95% CI 0.7285–0.7387) for the validation cohort, indicating good discriminative ability. The calibration curve showed a perfect fit between the predicted prediabetes risk and the observed prediabetes risk. An analysis of the decision curve presented the clinical application of the nomogram, with alternative threshold probability spectrums being presented as well. A personalized prediabetes prediction nomogram was developed and validated among Chinese adults, identifying high-risk individuals. Doctors and others can easily and efficiently use our prediabetes prediction model when assessing prediabetes risk.

## Introduction

Prediabetes is a condition characterized by a state of hyperglycemia, where blood sugar levels are higher than normal but lower than in diabetes^1^. According to 2013 estimates, 35.7% of Chinese adults had prediabetes^2^. Every year, approximately 5–10% of prediabetic people develop diabetes mellitus (DM), with 70% eventually becoming DM^3^. Prediabetic people have a higher risk of developing a number of complications related to diabetes, including microvascular complications like kidney, retina, and nervous system problems, as well as macrovascular complications like cardiovascular disease^4–6^. Moreover, the hyperglycemia status prior to the onset of diabetes can cause deterioration in the nervous system, kidneys, retina, and macro-vessels^7–9^. This has led to a significant burden of prediabetes-related diseases and disorders on families and society. Consequently, it is crucial to develop a screening tool that can accurately identify those with undiagnosed prediabetes or at high risk of developing it. This will help diabetes prevention programs be implemented effectively.

Pre-diabetes is a term used to describe the transitional phase from normal glucose metabolism to diabetes and encompasses impaired fasting glucose (IFG) and impaired glucose tolerance (IGT)^10^. In China, the diagnostic criteria for pre-diabetes defined by the American Diabetes Association (ADA) are commonly used. Accordingly, a fasting plasma glucose (FPG) level of 5.6–6.9 mmol/L is regarded as the threshold for IFG^11^. It is possible to maintain blood glucose levels in patients with prediabetes and even restore their health through artificial interventions^12^. According to some studies, lifestyle modification plays a significant role in diabetes prevention and can reduce relative risk by 40% to 70%^3^. Preventive interventions initiated during the pre-diabetes stage are more efficient and cost-effective than interventions initiated after the onset of diabetes. The reason is that they can delay or prevent the progression from prediabetes to diabetes^13,14^.

Factors influencing prediabetes include age^15,16^, marital status^15^, educational attainment^15^, hypertension^17^, dyslipidemia^18^, gestational diabetes, body mass index (BMI)^15,19^, waist circumference (WC)^19^, diet patterns^20^, and 1-h plasma glucose levels^21^. Using the risk score model, it becomes easier to assess individuals' prediabetes development status and screen out the high-risk population. The nomogram is an intuitive model for predicting the risk, providing accurate and individualized predictions for each person^22^. Many diabetes risk score models are now available to optimize diabetes risk estimation and make a diabetes risk assessment and patient intervention decisions^23–26^. Several risk assessment tools for detecting those with prediabetes have been reported^27,28^. However, most of these studies were cross-sectional and relied heavily on logistic regression analysis to develop the model. In addition, the majority of these models were developed for Caucasians in developed countries, and there are very few scoring systems available for Asians. A risk score developed from one ethnic group may not be applicable to another^29^. In view of this, a prediabetes risk score or nomogram should be developed for the Chinese adult population.

Several predictive models for prediabetes based on Chinese cohorts have emerged recently. Because of their small sample size, failure to use Cox proportional hazards models that take into account the factors of follow-up time to build the model, and lack of evaluation of model accuracy and clinical value of use, the model's generalization is somewhat limited^30,31^. Our research aimed to use Cox proportional hazards models to build a nomogram based on the data in the Chinese medical examination reports. Furthermore, we will thoroughly evaluate the model's discrimination, clinical utility, and calibration. Our prediabetes risk prediction model was designed to assist physicians in predicting prediabetes and developing related intervention plans to help patients prevent or delay its onset.

## Methods

### Study design

We followed the methods of Yong Han et al.^32^. We conducted a retrospective cohort study using data from the database provided by China Rich Healthcare Group. Baseline variables were selected as screening factors for the prediction model in this study. The dependent variable was incident prediabetes diagnosed during the five-year follow-up, represented as a dichotomous variable with 0 indicating non-prediabetes and 1 indicating prediabetes.

### Data source

The raw data used in this study was obtained from the DATADRYAD database (https://datadryad.org/stash), which was freely provided by Chen, Ying et al. (2018) in their publication "Association of body mass index and age with incident diabetes in Chinese adults: a population-based cohort study." Using this data for secondary analyses was permitted under Dryad's terms of service without violating the authors' rights.

### Study population

To minimize selection bias, participants who underwent a health examination were non-selectively and consecutively chosen from 32 locations and 11 cities in China, including Beijing, Guangzhou, Nanjing, Suzhou, Shanghai, Shenzhen, Changzhou, Nantong, Hefei, Chengdu, and Wuhan. Non-traceable codes were used to encode participants to ensure their privacy. Data were retrieved from the China Rich Healthcare Group electronic medical record system, and the original study was approved by the Rich Healthcare Group Review Board. Informed consent was waived due to the study's retrospective nature^33^.

**Figure 1:**
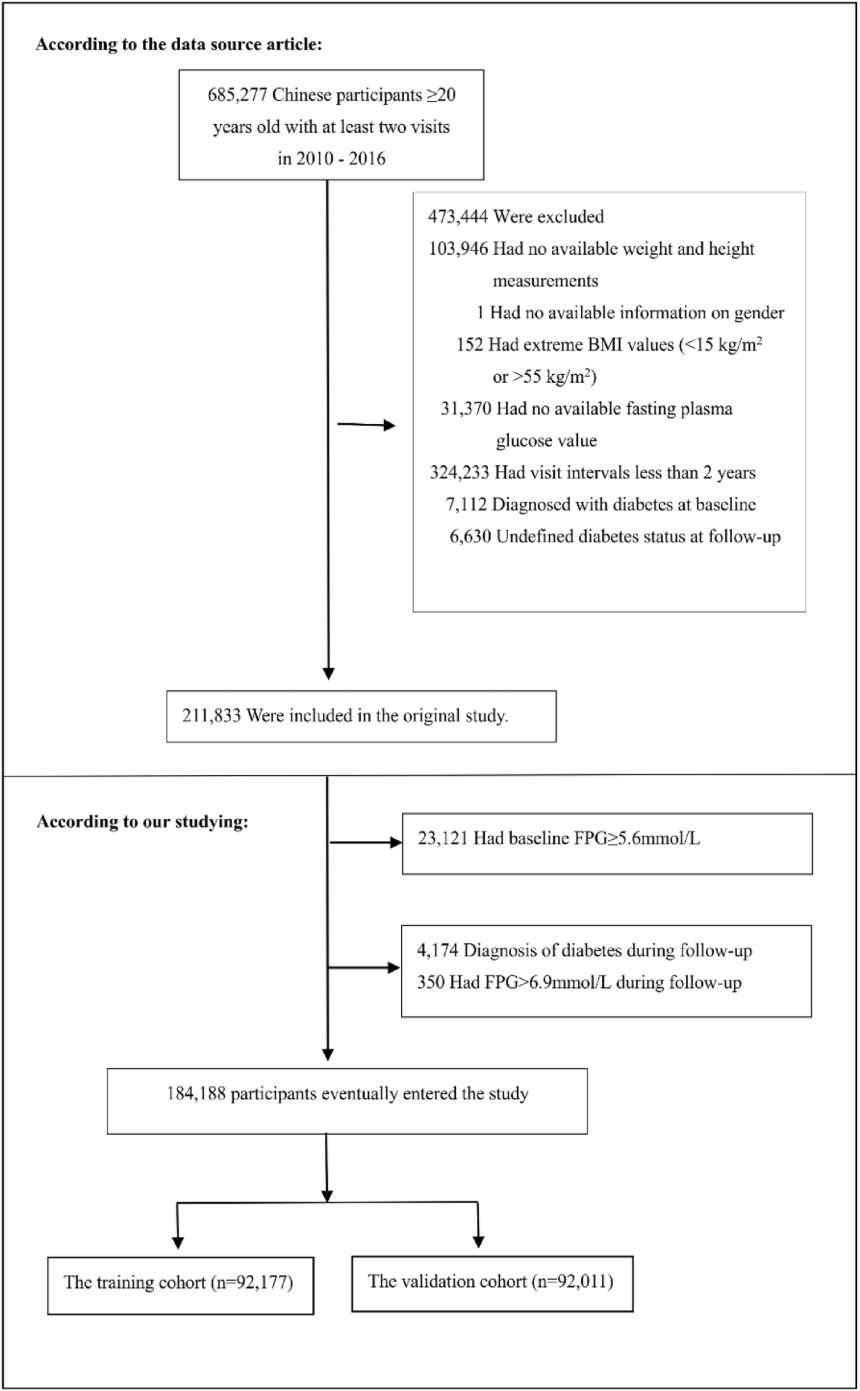
Flowchart of study participants. Figure showed the inclusion of participants. A total of 211,833 participants were assessed for eligibility in the original study. 27,645 participants were excluded, which left 184,188 subjects in the final analysis.

The study initially enrolled 685,277 participants, of whom 501,089 were excluded, leaving a final sample of 184,188 participants for analysis (as illustrated in Fig. 1). Eligible participants were those who had undergone health checks at least twice between 2010 and 2016. Exclusion criteria were established^34^ and included: (1) participants with missing information on baseline weight, FPG, gender, or height (n = 135,317); (2) those with a visiting period less than 2 years (n = 324,233); (3) individuals with extreme BMI values (< 15 kg/m2 or > 55 kg/m2) (n = 152); (4) those with unknown diabetes status at follow-up (n = 6,630); (5) participants diagnosed with diabetes at baseline (n = 7112); (6) individuals with self-reported diabetes or FPG ≥ 6.9 mmol/L during follow-up (n = 4524); and (7) those with baseline FPG ≥ 5.6 mmol/L (n = 23,121).

### Variables

#### Baseline variables

Based on previous research and clinical experience, we selected several variables as screening variables for the prediction model in this study^34^. The following variables were therefore used as screening variables based on the principles outlined above: (1) continuous variables: systolic blood pressure (SBP), age, low-density lipoprotein cholesterol (LDL-c), diastolic blood pressure (DBP), serum creatinine (Scr), total cholesterol (TC), alanine aminotransferase (ALT), high-density lipoprotein cholesterol (HDL-c), BMI, triglyceride (TG), FPG, blood urea nitrogen (BUN), aspartate aminotransferase (AST); (2) categorical variables: smoking status, family history of diabetes, gender, and drinking status.

During each visit to the health check center, participants were given a detailed questionnaire, which included questions about their lifestyle, demographic characteristics, family history of diabetes, and personal medical history. Trained staff measured the participants' weight, blood pressure, and height. Weight was measured with an accuracy of 0.1 kg while wearing light clothing and no shoes. Height was measured accurately to within 0.1 cm. BMI was calculated by dividing weight (kg) by height (m) squared. Blood pressure was measured using mercury sphygmomanometers. Participants fasted for at least 10 h before each appointment, and fasting venous blood samples were collected. HDL-c, Scr, AST, TC, FPG, BUN, TG, ALT, and LDL-c were measured on an autoanalyzer (Beckman 5800)^34^.

#### Handling of missing baseline variables

The number of participants with missing data of SBP, DBP, TC, TG, HDL-c, LDL-c, ALT, AST, BUN, Scr, smoking status, and drinking status was 16 (0.0087%), 17 (0.0092%), 4206 (2.28%), 4237 (2.30%), 83,351 (45.25%), 82,850 (44.98%), 1539 (0.84%), 107,655 (58.45%), 18,552 (10.07%), 9756 (5.30%), 133,209 (72.36%), and 133,209 (72.36%), respectively. Missing variables data were handled with multiple imputations^35^. The imputation model included BMI, SBP, age, HDL-c, gender, Scr, TC, ALT, DBP, TG, LDL-c, BUN, FPG, AST, family history of diabetes, drinking, and smoking status. The assumption of missing-at-random (MAR) is commonly employed in statistical analyses that deal with missing data^36^. Considering several variables among those selected for analysis exhibit significant missing data. For instance, smoking and alcohol consumption statuses, AST, and HDL-c. In order to validate whether their missingness is random, we divided the study population into two groups based on whether smoking consumption statuses, drinking consumption statuses, AST or HDL-c data were missing. By comparing the differences in age, gender, BMI and other indicators between the two groups, we analyzed whether the missingness of these variables was random. The comparison results showed that the differences between the two groups are relatively small (SD < 10%) for most indicators such as age, BMI, SBP, TG, FPG, and prediabetic incidence rate. This suggested that the missingness of data such as smoking and drinking status, AST, and HDL-c may be random (Table S1–S4).

### Outcome measures

Our interesting outcome variable was pre-diabetes (dichotomous variable: 0 = non-prediabetes, 1 = pre-diabetes). Prediabetes is diagnosed based on IFG, and according to the ADA's 2018 diagnostic criteria, FPG values in prediabetic patients are set at 5.6 to 6.9 mmol/L^11^. Participants were censored either upon diagnosis of prediabetes or at their last visit. A five-year follow-up period was used.

### Statistical analysis

The participants were randomly divided into two groups: the training and validation cohorts. Baseline characteristics of continuous variables were expressed as means with standard deviations or medians with quartiles for skewed distributions, while categorical variables were expressed as frequencies or percentages. Differences between the two cohorts were analyzed using t-tests for normally distributed continuous variables, Wilcoxon rank-sum tests for non-normally distributed variables, and chi-square tests for categorical variables. We also examined the training and validation cohorts' baseline characteristics stratified by incident prediabetes.

In order to find a reliable and simple risk prediction model, we established five models for comparison on the training cohorts. First, we applied all risk factors to build a full model through the Cox proportional hazards model. Second, an akaike information criterion (AIC)-based backward step-down approach was employed to develop a parsimonious model (stepwise Cox proportional hazards model)^37^. Third, in order to determine the significant variables and functional form, we used the multivariable fractional polynomials (MFP) algorithm in an iterative manner to establish a stable model (MFP model) in the real world^38^. The fourth method employed in this study is gradient tree boosting, which is implemented using the eXtreme Gradient Boosting (XGBoost) system. This machine-learning method is highly effective and involves assembling weak prediction models to establish a more reliable and accurate prediction model^39–41^. Therefore, we used the XGBoost system to develop a machine-learning model. Fifth, the least absolute shrinkage and selection operator (LASSO) regression is the first variable screening method since it is suitable for reducing high-dimensional data and selecting the most useful prediction candidates^42,43^. To establish the LASSO model, candidates with non-zero coefficients were selected^44^. We would examine the performance of the above five models and choose one that required the fewest variables, was simple and practical, and performed well for future analysis. At the same time, we validated the above five models on the validation set.

To evaluate the discriminatory power of the predictive model, we plotted and calculated the receiver operating characteristic (ROC) curve and the area under the ROC curve (AUC) with 95% confidence intervals for the training cohorts. The specificity, sensitivity, positive likelihood ratio (PLR), negative likelihood ratio (NLR), negative predictive value (NPV), and positive predictive value (PPV) were calculated according to standard definitions for the final model. Simultaneously, we validated the ROC curve and model performance in the validation cohort.

Besides, we obtained a prediabetic prediction formula from the final model. Predicted risk (time t) = 1-S_0_(t)^Exp(LP)^. The final selected model was used to predict the probability at time t (in years) after the start of follow-up. LP = linear predictor from the final model. Exp = exponential of e. S_0_ (t) = baseline survival at time t (for ease of calculation an estimate was provided five years after the start of follow-up)^45^.

The nomogram was created by proportionally converting each regression coefficient in the final selected model to a 0-to-100-point scale^46^. A 100-point score was assigned to the effect of the variable with the highest β coefficient (absolute value). We combined independent variables to derive total points, which were converted into predicted probabilities of developing prediabetes. Basically, the nomogram score represented the prediction model score for each patient.

Besides, an assessment of the nomogram's accuracy was performed using the calibration plot for five-year prediabetes probability^47^. For the purpose of determining the clinical utility of the prediabetes risk prediction model, a decision curve analysis was performed: the proportion of people showing true positive results minus the proportion of people showing false positive results, then calculated the net benefits of making a decision based on the relative hazard of false positives and false negatives^48^. It should be noted that we simultaneously evaluated the accuracy and clinical utility of the model in both the training cohort and the validation cohort.

We utilized the Kaplan–Meier method to calculate the survival estimates and time-to-event variables. In addition, we used the log-rank test to assess the likelihood of prediabetes-free survival among the four predicted probability of prediabetes groups.

We also analyzed the performance of each risk factor in the model for predicting prediabetes performance and its optimal cutoff using ROC curves. A DeLong test was used to compare the AUC of each risk factor. According to the TRIPOD statement, all results were reported^49^.

The statistical analyses were performed using R (http://www.R-project.org, The R Foundation) and Empower-Stats (X&Y Solutions, Inc, Boston, MA). All tests were two-tailed, and statistical significance was set at a P-value of less than 0.05.

### Ethics approval and consent to participate

The Rich Healthcare Group Review Board reviewed and approved studies involving human participants, and retrospective information was retrieved. It was conducted in accordance with the ethical principles of the Declaration of Helsinki. The data are anonymous, and the Rich Healthcare Group Review Board waived the requirement for informed consent due to the study's observational nature, as reported elsewhere^34^.

## Results

The study had 184,188 eligible participants (53.06% males and 46.94% females). The selection process of participants was shown in Fig. 1. Overall, the mean age of the participants was 41.02 ± 12.10. The median follow-up period was 3.00 years, and 19,699 (10.70%) participants developed prediabetes during that time. A mean BMI of 22.99 ± 3.26 kg/m^2^ was recorded. Mean SBP and DBP were 117.82 ± 15.81 and 73.53 ± 10.60 mmHg, respectively. Regarding FPG, the mean was 4.77 ± 0.49 mmol/L.

### Baseline characteristics of participants

A basic description of the demographics, anthropology, and clinical characteristics of the eligible participants was provided in Table 1. We divided all participants into the training cohort (n = 92,177) and the validation cohort (n = 92,011). The median follow-up period of the training and validation cohorts was 3.00 years, and 9859 and 9840 participants developed prediabetes, respectively. No statistically significant difference was observed among all baseline characteristics between the training and validation cohorts (all *P* > 0.05).

**Table 1:** Baseline characteristics of the training and validation sets.

Characteristic	Training set	Validation set	*P*-value
Participants	92,177	92,011	
Incident prediabetes			0.993
No	82,318 (89.3%)	82,171 (89.3%)	
Yes	9859 (10.7%)	9840 (10.7%)	
Age (year)	41.0 ± 12.1	41.0 ± 12.1	0.302
BMI (kg/m^2^)	23.0 ± 3.3	23.0 ± 3.3	0.439
SBP (mmHg)	117.8 ± 15.8	117.9 ± 15.9	0.198
DBP (mmHg)	73.5 ± 10.6	73.5 ± 10.6	0.574
FPG (mmol/L)	4.8 ± 0.5	4.8 ± 0.5	0.036
TC(mmol/L)	4.7 ± 0.9	4.7 ± 0.9	0.509
TG (mmol/L)	1.0 (0.7–1.5)	1.0 (0.7–1.5)	0.362
HDL-c (mmol/L)	1.4 ± 0.3	1.4 ± 0.3	0.959
LDL-c (mmol/L)	2.7 ± 0.7	2.7 ± 0.7	0.292
ALT (U/L)	17.5 (12.5–26.7)	17.5 (12.6–26.7)	0.405
AST (U/L)	22.0 (17.6–27.7)	22.0 (17.6–27.6)	0.990
BUN (mmol/L)	4.6 ± 1.2	4.6 ± 1.2	0.060
Scr (umol/L)	69.5 ± 15.2	69.6 ± 16.2	0.168
Gender			0.528
Male	48,843 (53.0%)	48,890 (53.1%)	
Female	43,334 (47.0%)	43,121 (46.9%)	
Smoking status			0.734
Current	14,920 (16.2%)	14,985 (16.3%)	
Ever	3215 (3.5%)	3164 (3.4%)	
Never	74,042 (80.3%)	73,862 (80.3%)	
Drinking status			0.535
Current	1545 (1.7%)	1486 (1.6%)	
Ever	11,524 (12.5%)	11,572 (12.6%)	
Never	79,108 (85.8%)	78,953 (85.8%)	
Family history			0.533
No	90,373 (98.0%)	90,173 (98.0%)	
Yes	1804 (2.0%)	1838 (2.0%)	

Values are n(%), mean ± SD, or medians (quartiles).

BMI, Body mass index; AST, Aspartate aminotransferase; SBP, Systolic blood pressure; TC, Total cholesterol; FPG; Fasting plasma glucose; DBP, Diastolic blood pressure; TG, Triglyceride; ALT, Alanine aminotransferase; HDL-c, High-density lipoprotein cholesterol; BUN, Blood urea nitrogen; LDL-c, Low-density lipid cholesterol; Family history, Family history of diabetes; Scr, Serum creatinine.

Table 2 displayed the baseline characteristics of the two cohorts based on their five-year incident prediabetes status. Participants who developed prediabetes during the study showed higher levels of SBP, TG, age, DBP, FPG, BMI, TC, ALT, Scr, LDL-C, AST, BUN, and a higher prevalence of males, ever or current smokers and drinkers in both the training and validation cohorts (all *P* < 0.01). Conversely, they had lower levels of HDL-C. Additionally, in the validation cohort, there was a higher proportion of participants with a family history of diabetes among those who developed prediabetes compared to those who did not. However, there was no statistically significant difference in the family history of diabetes in the training cohort (*P* = 0.054).

**Table 2:** Baseline characteristics for the training and validation cohorts by incident prediabetes status.

Characteristic	Training cohort			Validation cohort		
	Non-diabetes	Prediabetes	*P* value	Non-diabetes	Prediabetes	*P* value
Participants	82,318	9859		82,171	9840	
Age (year)	40.3 ± 11.7	46.9 ± 13.7	< 0.001	40.3 ± 11.7	46.5 ± 13.5	< 0.001
Gender			< 0.001			< 0.001
Male	42,466 (51.6%)	6377 (64.7%)		42,629 (51.9%)	6261 (63.6%)	
Female	39,852 (48.4%)	3482 (35.3%)		39,542 (48.1%)	3579 (36.4%)	
BMI (kg/m^2^)	22.8 ± 3.2	24.3 ± 3.3	< 0.001	22.8 ± 3.2	24.4 ± 3.3	< 0.001
SBP (mmHg)	117.0 ± 15.4	124.4 ± 17.1	< 0.001	117.0 ± 15.5	124.6 ± 17.3	< 0.001
DBP (mmHg)	73.1 ± 10.4	77.2 ± 11.2	< 0.001	73.1 ± 10.4	77.3 ± 11.2	< 0.001
FPG (mmol/L)	4.7 ± 0.5	5.0 ± 0.4	< 0.001	4.7 ± 0.5	5.0 ± 0.4	< 0.001
TG (mmol/L)	1.0 (0.7–1.5)	1.3 (0.9–1.9)	< 0.001	1.0 (0.7–1.5)	1.3 (0.9–1.9)	< 0.001
HDL-C(mmol/L)	1.4 ± 0.3	1.3 ± 0.3	< 0.001	1.4 ± 0.3	1.3 ± 0.3	< 0.001
LDL-C(mmol/L)	2.7 ± 0.7	2.8 ± 0.7	< 0.001	2.7 ± 0.7	2.8 ± 0.7	< 0.001
TC(mmol/L)	4.6 ± 0.9	4.8 ± 0.9	< 0.001	4.6 ± 0.9	4.9 ± 0.9	
ALT(U/L)	17.0 (12.2–26.0)	21.0 (14.9–32.0)	< 0.001	17.0 (12.3–26.0)	21.0 (14.9–32.0)	< 0.001
AST(U/L)	21.9 (17.4–27.4)	23.5 (19.0–29.8)	< 0.001	21.8 (17.5–27.3)	23.5 (19.0–29.7)	
BUN (mmol/L)	4.6 ± 1.2	4.8 ± 1.2	< 0.001	4.6 ± 1.2	4.8 ± 1.2	< 0.001
Scr (umol/L)	69.2 ± 15.2	72.7 ± 15.3	< 0.001	69.3 ± 16.3	72.7 ± 15.5	< 0.001
Smoking status			< 0.001			< 0.001
Current	12,776 (15.5%)	2144 (21.7%)		12,838 (15.6%)	2147 (21.8%)	
Ever	2795 (3.4%)	420 (4.3%)		2789 (3.4%)	375 (3.8%)	
Never	66,747 (81.1%)	7295 (74.0%)		66,544 (81.0%)	7318 (74.4%)	
Drinking status			< 0.001			< 0.001
Current	1293 (1.6%)	252 (2.6%)		1212 (1.5%)	274 (2.8%)	
Ever	9993 (12.1%)	1531 (15.5%)		10,026 (12.2%)	1546 (15.7%)	
Never	71,032 (86.3%)	8076 (81.9%)		70,933 (86.3%)	8020 (81.5%)	
Family history			0.054			< 0.001
No	80,732 (98.1%)	9641 (97.8%)		80,581 (98.1%)	9592 (97.5%)	
Yes	1586 (1.9%)	218 (2.2%)		1590 (1.9%)	248 (2.5%)	

Values are n(%), mean ± SD, or medians (quartiles).

BMI, Body mass index; AST, Aspartate aminotransferase; SBP, Systolic blood pressure; TC, Total cholesterol; FPG; Fasting plasma glucose; DBP, Diastolic blood pressure; TG, Triglyceride; ALT, Alanine aminotransferase; HDL-c, High-density lipoprotein cholesterol; BUN, Blood urea nitrogen; LDL-c, Low-density lipid cholesterol; Family history, Family history of diabetes; Scr, Serum creatinine.

### Univariate and multivariate analysis

Based on a univariate and multivariate Cox proportional hazards model in the training cohort, Table S5 showed risk factors for incident prediabetes. The univariate analysis showed that age (HR = 1.034), female (HR = 0.622), BMI (HR = 1.121), SBP (HR = 1.025), DBP (HR = 1.029), FPG (HR = 5.728), TG (HR = 1.186), LDL-C (HR = 1.244), HDL-C (HR = 0.733), ALT (HR = 1.004), AST (HR = 1.006), BUN (HR = 1.145), Scr (HR = 1.015), ever smoking (HR = 0.894), never smoking (HR = 0.700), ever drinking (HR = 0.784), and never drinking (HR = 0.599) were associated with incident prediabetes (all *P* < 0.05), family history of diabetes was not associated with prediabetes (P = 0.773). The multivariate analysis showed that age (HR = 1.020), female (HR = 0.918), BMI (HR = 1.047), SBP (HR = 1.008), DBP (HR = 1.003), FPG (HR = 4.611), TG (HR = 1.067), HDL-C (HR = 1.214), LDL-C (HR = 0.947), ALT (HR = 1.003), BUN (HR = 0.967), and Scr (HR = 1.006) were associated with incident prediabetes (all *P* < 0.05). However, AST, smoking and drinking status were not associated with prediabetes (all *P* > 0.05).

### Comparison of different models

**Figure 2:**
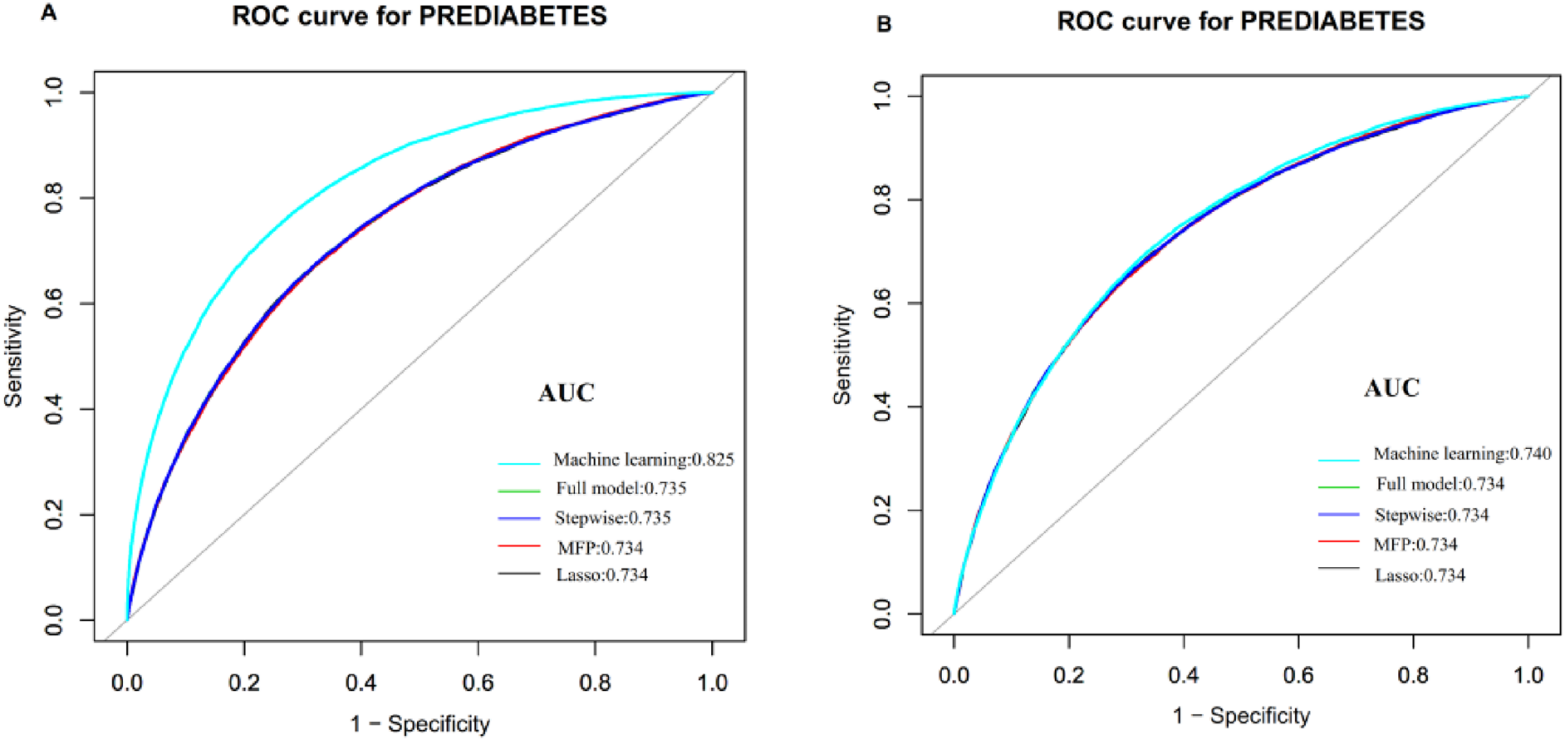
Comparison of the AUC of different models. (**A**) In the training set, the AUCs of the machine learning, LASSO, full, stepwise, and MFP models were 0.8252, 0.7341, 0.7351, 0.7350 and 0.7343, respectively. (**B**) In the validation set, the results of the validation suggested the corresponding AUCs of those models were 0.7403, 0.7336, 0.7343, 0.7342, and 0.7341, respectively.

We established five prediction models, including the stepwise, full, MFP, machine learning, and LASSO models. We screened among 17 candidate variables (BMI, SBP, age, HDL-c, gender, Scr, TC, ALT, DBP, family history of diabetes, TG, LDL-c, BUN, FPG, AST, drinking, and smoking status) employing the five different models. The above 17 variables are included in the full model except for TC. 14 variables from the data transformation were included in the MFP model, 13 in the stepwise model, and 17 in the machine learning model. While the LASSO model only included 6 variables. In the training cohort, AUCs of the machine learning, LASSO, full, stepwise, and MFP models were 0.8252, 0.7341, 0.7351, 0.7350 and 0.7343 (Fig. 2A). In the validation cohort, we validated the above 5 models. AUCs of the machine learning, LASSO, full, stepwise, and MFP models were 0.7403, 0.7336, 0.7343, 0.7342 and 0.7341, respectively (Fig. 2B). The AUC of these five models was relatively close. The machine learning model, although having the largest AUC in the training cohort, was somewhat inconvenient for practical clinical application given the large difference in AUC between the development and validation groups and the unavailability of a formula or nomogram. Since the LASSO model was able to predict the 5-year prediabetes risk accurately and had fewer risk factors incorporated, we opted to use it as the final prediction model for prediabetes.

### Identification of risk factors

**Figure 3:**
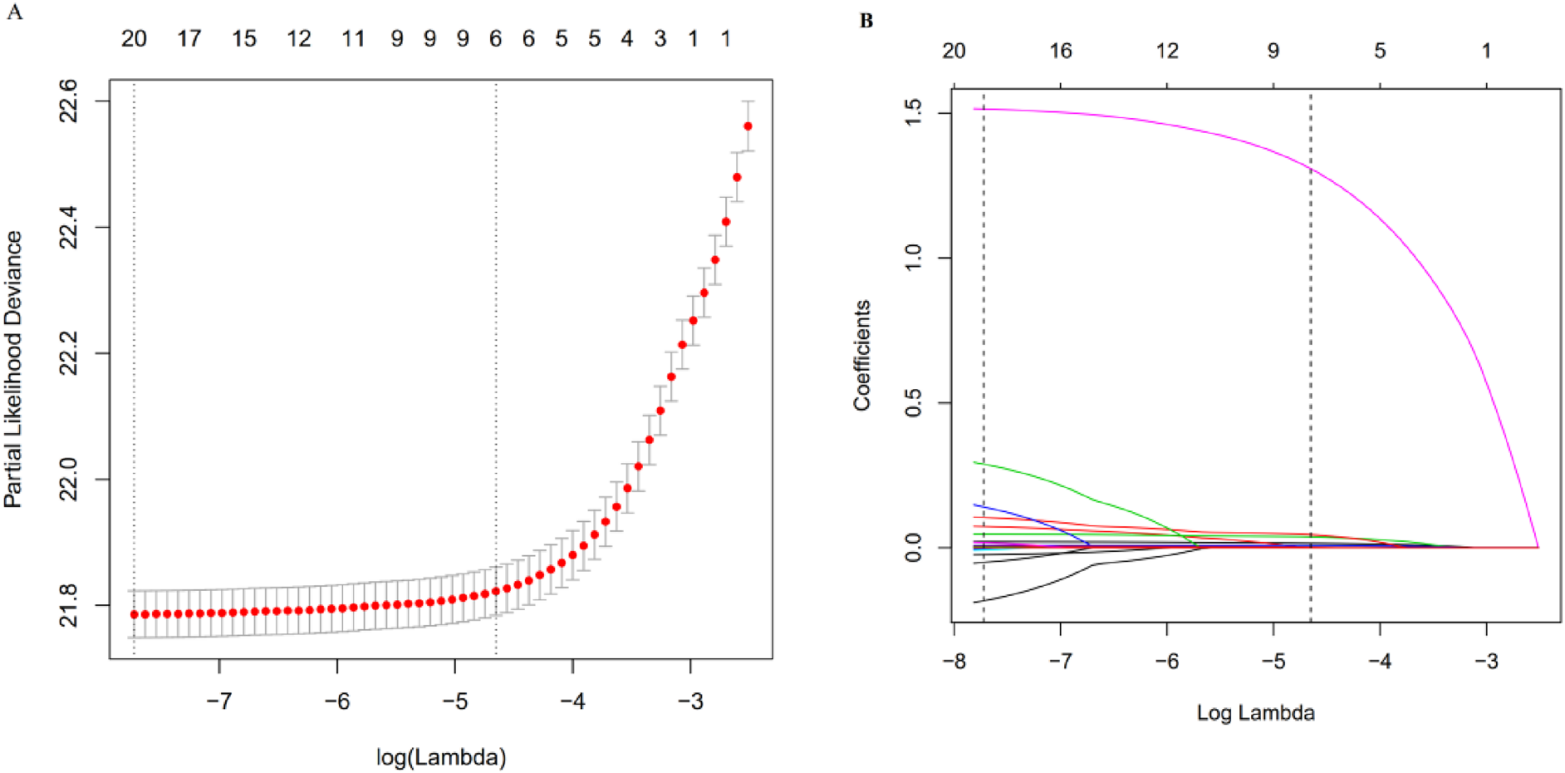
Risk predictors selection using the LASSO regression model. (**A**) Optimal predictor (lambda) selection in the LASSO model with tenfold cross-validation by minimum criteria. The area under the receiver operating characteristic curve was plotted versus log (lambda). Dotted vertical lines were drawn at the optimal values by using the minimum criteria and the 1 SE of the minimum criteria; (**B**) The LASSO coefficient profiles of the 17 predictors were shown. A coefficient profile plot was developed against the log (lambda) sequence. A vertical line was drawn at the value selected with tenfold cross-validation, resulting in 6 predictors with nonzero coefficients (lambda = 0.0095).

Out of 17 clinical features, only 6 potential predictors with non-zero coefficients in the LASSO regression model were identified based on data from 92,177 participants in the training set, as shown in Fig. 3A,B. These potential predictors were age, BMI, FPG, TG, Scr, and SBP. The study population ranged in age from 20 to 96 years old. BMI ranged from 15.0 to 45.64 kg/m^2^. FPG levels ranged from 0.59 to 5.59 mmol/L. TG levels ranged from 0.01 to 24.30 mmol/L. Scr ranged from 19.17 to 1116.60 μmol/L. SBP had a wide distribution from 66 to 222 mmHg. Table 3 showed the LASSO model selected the 6 variables, including FPG (HR 4.5577, 95% CI 4.3363–4.7903), BMI (HR 1.0494, 95% CI 1.0427–1.0561), age (HR 1.0184, 95% CI 1.0168–1.0199), TG (HR 1.0687, 95% CI 1.0519–1.0859), SBP (HR 1.0097, 95% CI 1.0084–1.0109) and Scr (HR 1.0062, 95% CI 1.0049–1.0075). The results showed that the six variables were all positively associated with incident prediabetes.

**Table 3:** Variables selected using Lasso regression model.

Variable	Beta	Standard error	HR (95% CI)	*P* value
FPG (mmol/L)	1.5168	0.0254	4.5577 (4.3363, 4.7903)	< 0.0001
Age (years)	0.0182	0.0008	1.0184 (1.0168, 1.0199)	< 0.0001
BMI (kg/m^2^)	0.0482	0.0033	1.0494 (1.0427, 1.0561)	< 0.0001
SBP (mmHg)	0.0096	0.0006	1.0097 (1.0084, 1.0109)	< 0.0001
TG (mmol/L)	0.0665	0.0081	1.0687 (1.0519, 1.0859)	< 0.0001
Scr (umol/L)	0.0062	0.0007	1.0062 (1.0049, 1.0075)	< 0.0001

FPG; Fasting plasma glucose; SBP, Systolic blood pressure; BMI, Body mass index; TG, Triglyceride; Scr, Serum creatinine; HR, Hazard ratios; CI, Confidence interval.

We demonstrated the prediction performance of each risk factor for 5-year incident prediabetes in the training and validation cohorts (Table S6, Figure S1A, S1B). FPG had the highest AUC among all the risk factors, significantly greater than other risk factors (*P* < 0.001).

**Figure 4:**
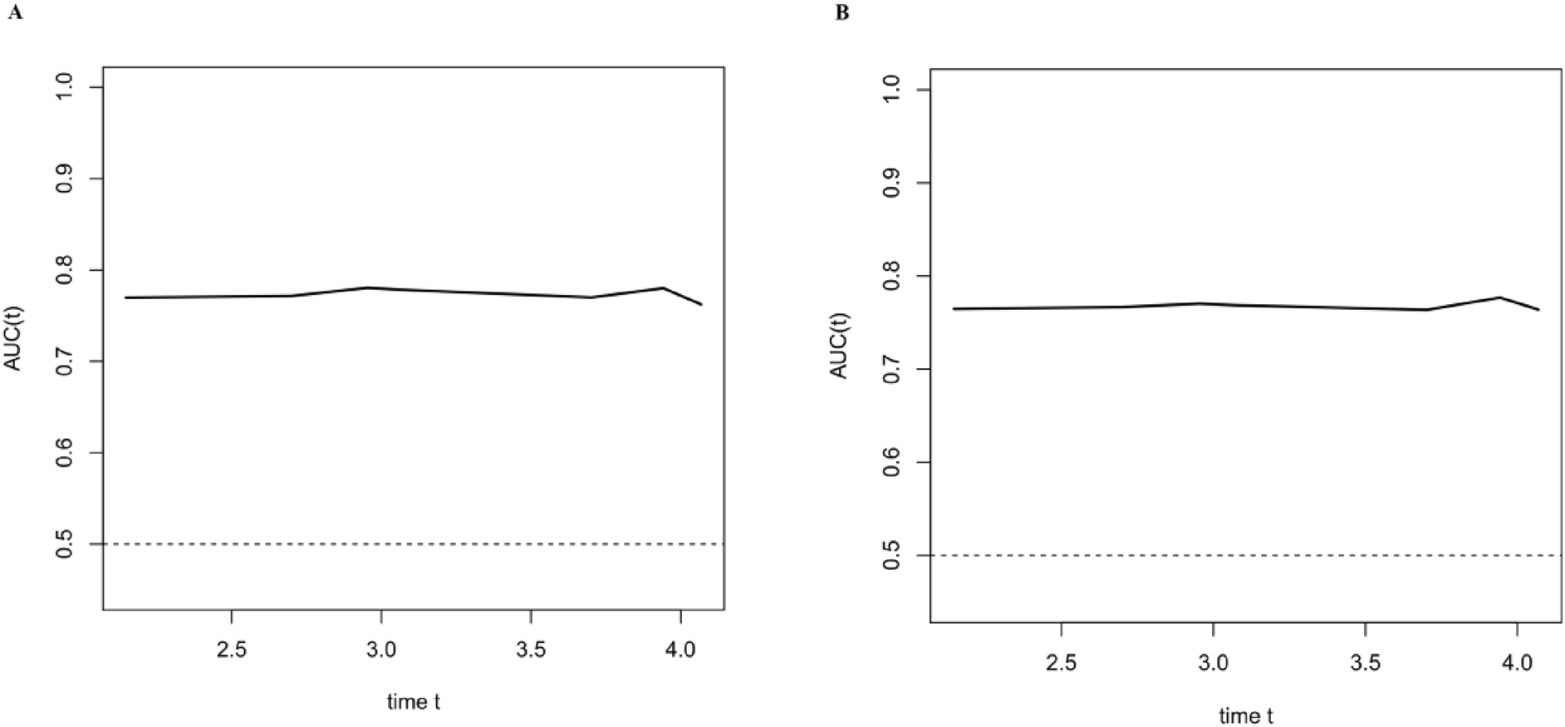
Time-dependent ROC curve. We plotted the time-dependent ROC curves for the LASSO model in the training cohort (**A**) and validated it in the validation cohort (**B**). The curves demonstrated that using the present model, the AUCs for predicting the risk of incident prediabetes at various future time points remained relatively stable. This indicates that the model has a strong and consistent predictive value for all cases of incident prediabetes at different future time points.

In addition, we generated time-dependent ROC curves for the LASSO model in the training and cohorts (Fig. 4A). Simultaneously, in the validation cohort, we validated the time-dependent ROC curve (Fig. 4B). These curves showed that the AUCs for predicting the risk of prediabetes at various future time points using the LASSO model remained consistent. This suggests that the LASSO model has good predictive value for incident prediabetes at different time points in the future.

### Development of the nomogram

**Figure 5:**
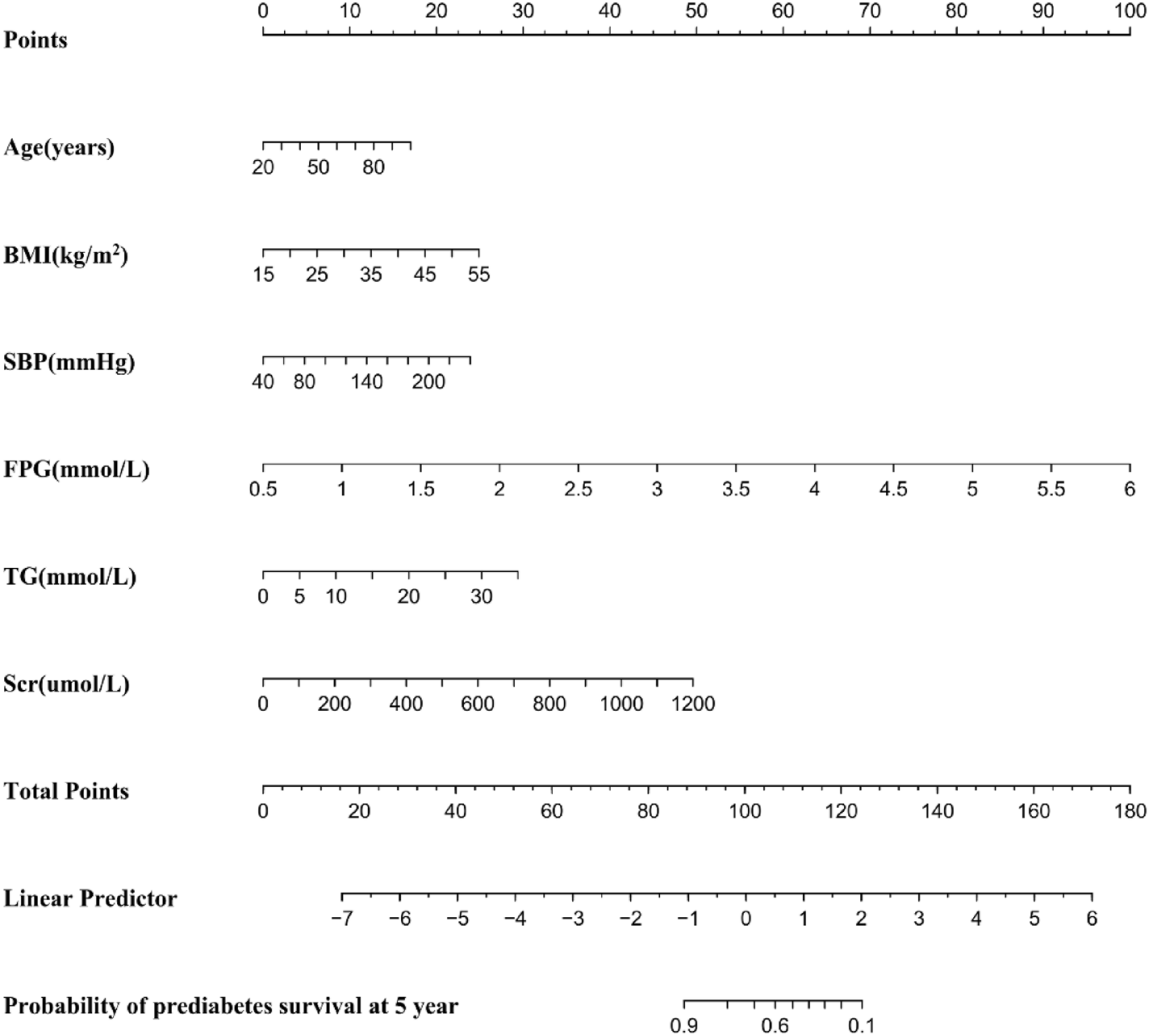
Nomogram to predict the risk of prediabetes for Chinese adults. Each risk predictor's score is plotted on the appropriate scale. A vertical line is drawn from each patient's score on the appropriate scale to the top points scale in order to determine the patient's score for each risk predictor. All scores are summed to obtain the total points score. Using the bottom portion of the total points scale, we can predict the probability of prediabetes occurring.

A corresponding nomogram was also created to provide a simple and quantitative way of predicting the development of prediabetes within five years using age, BMI, FPG, TG, SBP, and Scr (Fig. 5). Points were assigned for each variable value of the nomogram, and the sum of the points for each variable value was obtained. A five-year probability of prediabetes risk was calculated using this method. And the algorithm of prediabetes risk was as follows: Predicted risk (5-year) = 1-S_0_ (5-year)^Exp(LP)^. LP = 1.51681 * FPG (mmol/L) + 0.01820 * age (years) + 0.04821 * BMI (kg/m^2^) + 0.00963 * SBP (mmHg) + 0.06648 * TG (mmol/L) + 0.00617 * Scr (umol/L). S_0_ (5-year) = 0.999993.

### Prediction performance of the nomogram

#### Discrimination

The AUC of the nomogram in the training cohort was 0.7341 (95% CI 0.7290–0.7392). And the validation cohort. In the validation of the model, we found that the AUC was 0.7336 (95% CI 0.7285–0.7387) (Table 4, Fig. 2). According to the best threshold, the sensitivity rates for the training and validation cohorts were 68.00% and 65.35%, and the specificity rates were 67.13% and 69.91%. Notably, there was a relatively high NPV in both the training and validation cohorts.

**Table 4:** Prediction performance of the nomogram for the risk of prediabetes.

	AUC	95% CI		Best threshold of predicted prediabetes probability	Specificity (%)	Sensitivity (%)	PPV (%)	NPV (%)	PLR	NLR
		Lower	Upper							
Training cohort	0.7341	0.7290	0.7392	0.3537	67.13	68.00	20.74	94.31	2.069	0.477
Validation cohort	0.7336	0.7285	0.7387	0.3720	69.91	65.35	21.52	94.11	2.172	0.496

AUC, Area under the curve; CI, Confidence interval; NPV, Negative predictive value; PPV, Positive predictive value; NLR, Negative likelihood ratio; PLR, Positive likelihood ratio;

#### Model accuracy evaluation

**Figure 6:**
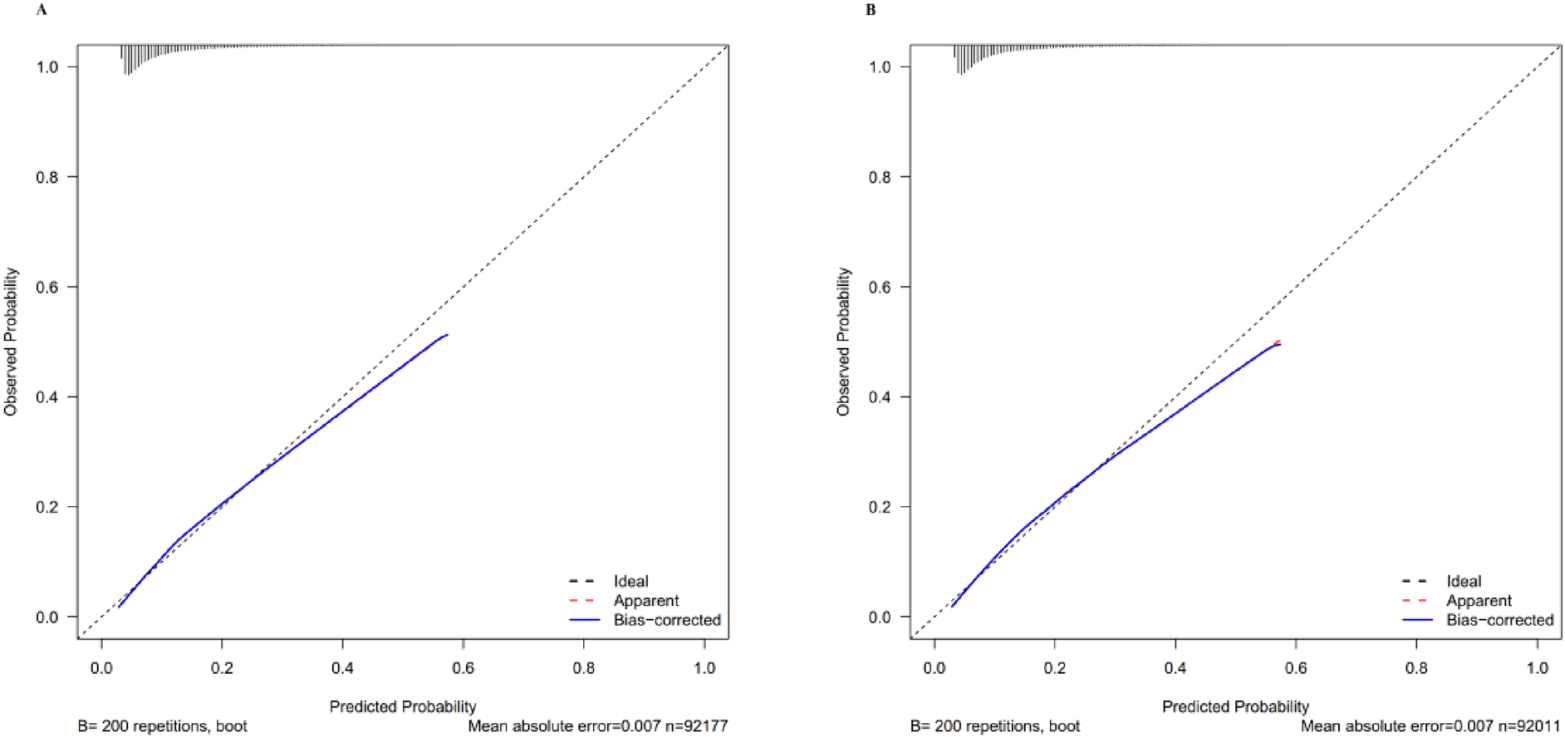
Calibration curves. It was found that the calibration curves for the 5-year probability of incident prediabetes demonstrated excellent agreement between the predicted probability and the actual observation in both training and validation sets (**A**, **B**). According to these results, the nomogram accurately predicted 5-year incidences of prediabetes in Chinese adults.

Furthermore, we determined whether the 5-year prediabetes risk predicted by the nomogram matched the observed 5-year risk in the training cohorts. At the same time, we also need to validate the model's accuracy in the validation cohort. Both training and validation sets of calibration curves showed excellent agreement between predicted possibilities and actual observations (Fig. 6A,B). According to these results, a nomogram could accurately predict the five-year incidence of prediabetes in a Chinese population.

### Clinical use of the nomogram

**Figure 7:**
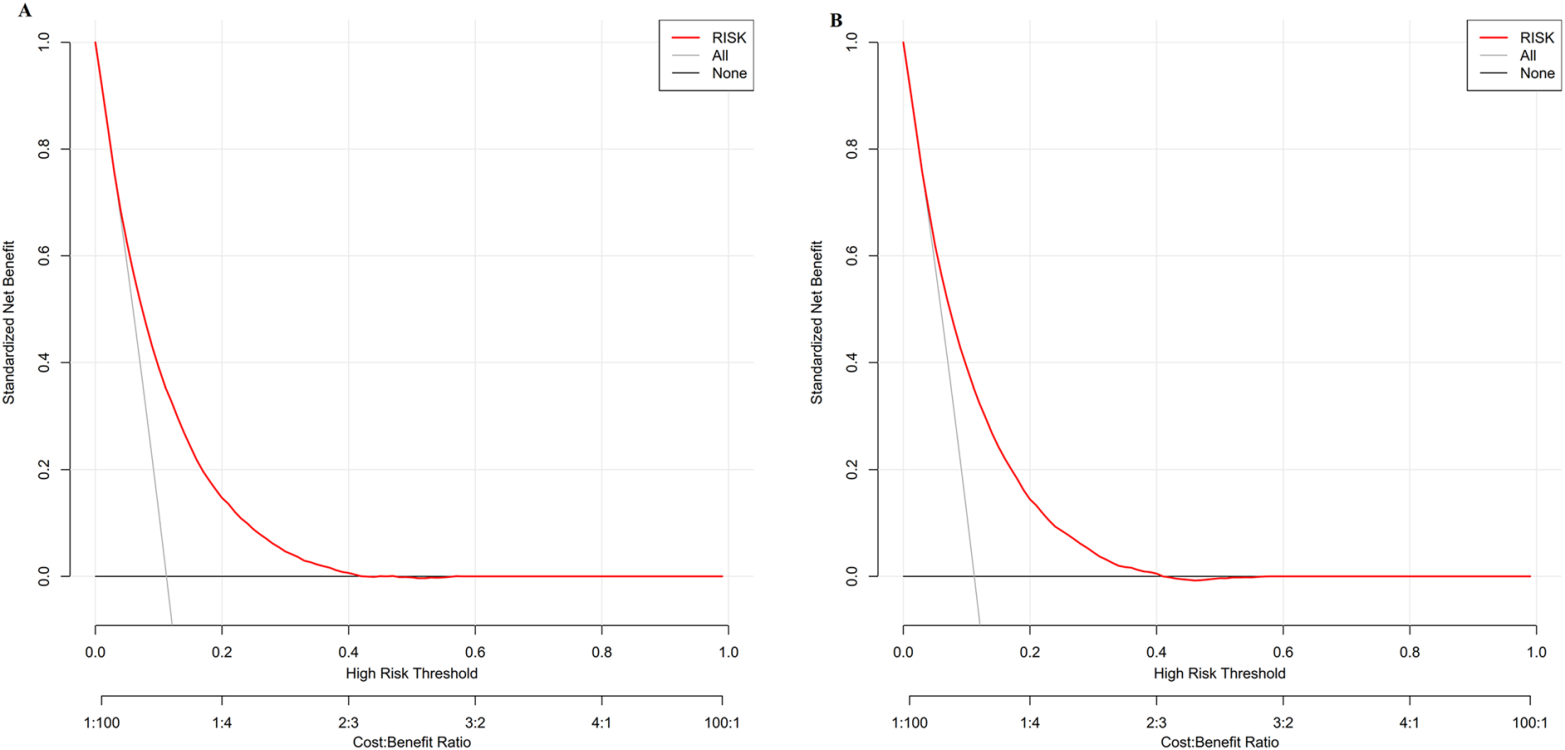
The decision curve analysis of the nomogram model for 5-year prediabetes risk in the training cohort (**A**) and validation cohort (**B**). When no participant is thought to acquire prediabetes, the black line shows the net benefit. When prediabetes is considered for all participants, the light gray line represents the net benefit. A model's clinical utility is indicated by the area between the “no treatment line” (black line) and the "all treatment line" (light gray line). The more distance between the model curve and the black and light gray lines, the better the nomogram's clinical value.

Figure analyzed the LASSO model's decision curves in the training cohorts. Figure 7B represents the results of the clinical decision curve validated in the validation cohort. The black line represented the net benefit, assuming that none of the participants developed prediabetes. In contrast, light gray lines were net benefits when prediabetes was considered for all participants. The area between the black line (no treatment line) and the light gray line (all treatment lines) in the model curve showed the model's clinical utility. In general, the farther the nomogram curve was from the black and light gray lines, the better its clinical utility. As an example, if a patient's threshold probability was 17% in the LASSO model, the net benefit would be about 20%, which was equivalent to performing 20 additional prediabetes screenings (such as oral glucose tolerance tests) per 100 Chinese adults when without a significant change in the incidence of prediabetes.

### Associations between predicted prediabetes probability and 5-year incident prediabetes

We divided both the training and validation cohorts into two groups based on whether they developed prediabetes in the future or not. We then compared the predicted probability of prediabetes at baseline between these two groups. The results revealed that participants who developed prediabetes had a higher predicted probability, while those who did not develop prediabetes had a lower predicted probability (Figure S2A, S2B).

**Figure 8:**
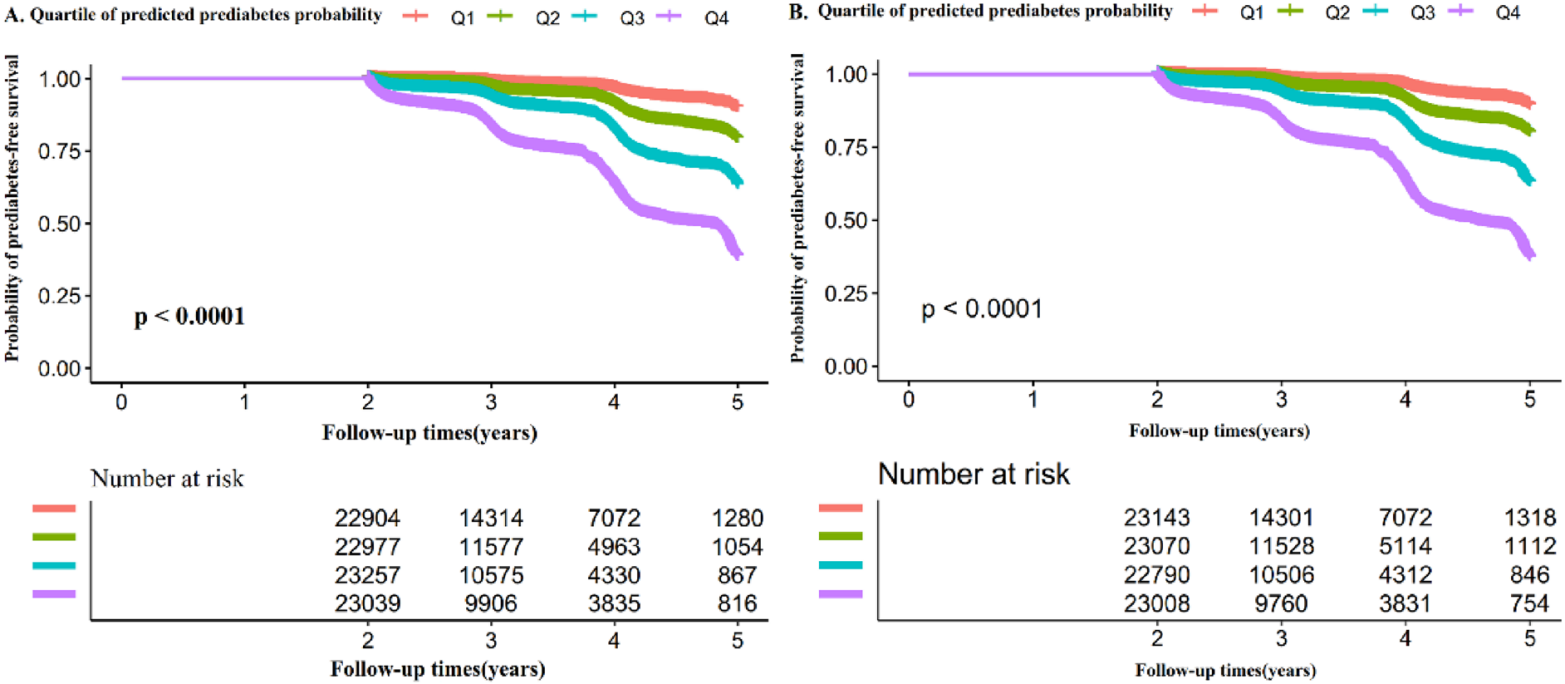
Kaplan–Meier event-free survival curve. Kaplan–Meier event-free survival curve in the training cohort (**A**) and validation cohort (**B**). We divided the participants into four groups based on the quartiles of predicted prediabetes probability at baseline. Kaplan–Meier survival curves for 5-year prediabetes-free survival probability stratified by the predicted probability groups. There were significant differences in the probability of prediabetes-free survival between the different predicted probability groups (log-rank test, *P* < 0.0001). Prediabetes-free survival probabilities decreased as predicted probability increased, which indicated that those with the highest predicted probability faced the highest risk of prediabetes.

We then stratified the participants into four groups based on the quartiles of predicted prediabetes probability at baseline. The Kaplan–Meier survival curves for 5-year prediabetes-free survival probability were plotted and stratified by the predicted probability groups (Fig. 8A,B). The results showed significant differences in the probability of prediabetes-free survival between the different predicted probability groups (log-rank test, *P* < 0.0001). As the predicted probability increased, the probability of prediabetes-free survival decreased, indicating that individuals with the highest predicted probability were at the greatest risk of developing prediabetes. These findings demonstrated the excellent performance of the LASSO model.

### Validation of the nomogram in participants with BMI ≥ 24 kg/m^2^

Additionally, considering BMI as an important risk factor for prediabetes and obesity as a high-risk population for prediabetes, we would validate our model in the population with BMI ≥ 24 kg/m^2^ based on the available data. According to the results, our model also demonstrated good performance in the population with BMI ≥ 24 kg/m^2^ (Table S7, Figure S3, S4). Therefore, this also further suggested that our model has a certain generalization value.

## Discussion

This study developed and validated a personalized prediction nomogram predicting 5-year incident prediabetes by cost-effective and readily available parameters among Chinese adults, which could be a tool for clinicians to identify high-risk individuals for prediabetes. The prediction model included six parameters: age, FPG, BMI, Scr, SBP, and TG. The model evaluation, and internal validation revealed that our nomogram performed exceptionally well in terms of prediction.

Although several risk assessment tools for detecting those with prediabetes have been reported^27,28,50–53^. A cross-sectional study from the Middle East established the Prediabetes Risk Score by sex, waist circumference (WC), age, BMI, and blood pressure. The AUC of the score was 80%, and the cut-off point of 16 yielded sensitivity and specificity of 86.2% and 57.9%, respectively^50^. Another study from the National Health and Nutrition Examination Survey (NHANES) developed a predictive model for pre-diabetes by age, BMI, waist circumference, history of high blood glucose, antihypertensive drug use, daily physical activity, family history of diabetes, and fruit & vegetable intake. The sensitivity and specificity of using the model (cutoff of ≥ 9) was 60.2% and 61.4% for pre-diabetes^53^. However, most of the studies were cross-sectional and relied heavily on logistic regression analysis to develop the model. Furthermore, the majority of these models were created for Caucasians in developed countries. Only a few reliable prediabetes prediction models were established in the Chinese population, including different risk predictors. Besides, the incidence rate of prediabetes, their prediction performance, and clinical usefulness varied greatly. In 2016, Ouyang Peng et al.^30^ developed a risk score using binary logistic regression analysis to predict the risk of prediabetes based on factors such as age, history of hypertension, BMI, DBP, family history of diabetes, and TG. The AUC of their model was 0.713 (95% CI 0.686 to 0.740). However, when screening the variables, they did not consider the FPG, BUN, Scr, ALT, and AST. Studies have shown that these variables contribute to prediabetes or diabetes^54–57^. Furthermore, the authors did not conduct a decision curve analysis to assess the clinical utility of the model, nor a calibration curve analysis to assess the model's accuracy. Additionally, a comparison and screening of other methods for incident prediabetes risk prediction were not performed. After all, screening variables directly using logistic regression models is not a good alternative, given the inherent colinearity and interaction effects between the screening variables. Furthermore, it is critical to consider the effect of follow-up time on outcomes for predictive models, as there may be differences in model prediction performance due to different follow-up times. Furthermore, age, BMI, TG, and DBP are continuous predictors of risk. Categorizing them into groups will result in a loss of information and a reduced ability to detect real relationships^58,59^. In 2021, Jiahua Wu et al.^31^ developed a model to predict the risk of prediabetes in middle-aged and elderly populations in China based on WC, HbA1c, family history of diabetes, and FPG. The AUCs were 0.702. Consistent with our nomogram, they also screened variables using the Cox proportional hazards model. However, the study did not establish time-dependent ROC curves or explicitly propose specific timing for predicting the risk of prediabetes. Moreover, the authors did not perform a decision curve analysis to evaluate the clinical utility of the model or assess the calibration of predicted risk against actual risk. Furthermore, they did not conduct internal and external validation of the prediction model, which may restrict the generalizability of their findings. The New Chinese Diabetes Risk Score (NCDRS), developed in 2013, provides a suitable risk measurement for type 2 diabetes mellitus (T2DM)^60^. NCDRS is a DM risk assessment that includes age, gender, WC, BMI, SBP, and family history of DM^60^. In 2020, Tao Mao et al.^61^ validated the predictive value of NCDRS in prediabetes. Because the NCDRS included relatively few risk predictors and did not include laboratory indicators, the prediabetes model may have insufficient accuracy and prediction performance. Thus, their model's predictive ability was relatively low, AUC = 0.694 (95% CI 0.683–0.705). In order to ensure prediction accuracy, we need to incorporate relatively more risk factors into the risk prediction model. It is worth pointing out that the sample sizes of the models mentioned above are relatively small. Our nomogram filled these gaps compared to the similar studies discussed above. Considering the size of the sample (n = 184,188) and the fact that participants were from multiple centers, our findings may be more applicable to Chinese individuals. In our screening process, we utilized machine learning, LASSO regression, and the multivariate fractional polynomials algorithm to account for collinearity and interaction among variables. Additionally, we established predictive equations using LASSO regression models to capture the impact of follow-up time on incident prediabetes and constructed time-dependent ROC curves. We conducted a thorough evaluation of the model for clinical usefulness, discrimination, and calibration, as well as internal validation. Our nomogram employs continuous variables to more precisely and individually predict risks.

Diabetes causes numerous complications, as well as severe physical and psychological distress for patients and a financial burden on the healthcare system. Because there are no specific symptoms, it is often undiagnosed. It may, however, be possible to increase screening yields and economic efficiency through oral glucose tolerance tests^62^. This study used the LASSO model with relatively good predictive performance to construct the nomogram. And using the risk predictors, we developed a formula to calculate prediabetes risk, which clinicians could use to identify high-risk individuals accurately. Since our nomogram items are routine clinical variables available to clinicians, clinicians can easily adopt them. It can also guide them in timing prediabetes screenings and reduce the time and effort spent on prevention and treatment for those at low risk for prediabetes. Furthermore, both training and validation groups showed high predictive performance, indicating good generalizability. In addition, it must be noted that the incidence of prediabetes in our study population was lower than in other similar studies (10.7% vs. 20.0–26.3%)^30,31^. A closer analysis of the relevant indicators revealed higher levels of age and BMI in their study population, as well as a higher proportion of family history of diabetes, smoking, and alcohol consumption. Studies have shown that these indicators are all critical influencing factors for diabetes or prediabetes^15,16,19,63–65^. Therefore, it is not surprising that the incidence of prediabetes was lower in our study population. It is known that the prevalence of the disease affects the positive and negative predictive values of diagnostics^66^. The comparatively low positive predictive value resulted from a low disease prevalence^67^. PPV increased with a rise in target disease prevalence when sensitivity and specificity were constant^61^.

The present study has the following strengths: (1) This study benefits from a large sample size and participants recruited from multiple institutions. (2) We developed five different prediction models: LASSO, full, stepwise, machine learning, and MFP. (3) We performed a nomogram to ensure the precision and clinical utility of the model. (4) Using risk predictors, we developed a formula to help clinicians quickly and accurately calculate the risk of prediabetes in individuals. Other similar studies can be verified externally with this information. (5) We perform a complete evaluation of the model for clinical use, discrimination, and calibration. (6) The results were validated to ensure reliability.

Despite the good performance of the nomogram, the study has some potential limitations. First of all, this was a second retrospective study. There were no other prediabetes risk factors in the raw data, such as medical history, waist/hip ratio, and lifestyle factors. However, despite the large sample size and participants from multiple centers, this study demonstrates excellent prediction performance in both training and validation groups, indicating high generalizability of the nomogram based on the existing six risk factors. Second, no oral glucose tolerance test or glycosylated hemoglobin measurements were conducted. According to one study, 55% of Asian diabetics were diagnosed based on FPG alone^68^. Hence, neglecting the consideration of IGT may overlook its potential impact on predicting the development of diabetes. In the future, we can consider designing our studies or collaborating with other researchers, to conduct an oral glucose tolerance test for all participants. Therefore, we can use the two criteria of IFG and IGT to diagnose the states of prediabetes, which will make our assessment of prediabetes more scientific. Third, although we used multiple imputations to replace missing values, this could still lead to bias as some variables had missing values of up to 50% or even more than 70%. For instance, smoking and alcohol consumption statuses are both missing over 70%, AST is missing 58.45%, and HDL-c is missing 45.25%. Fourth, due to the significant variations in dietary habits and a partial familial predisposition observed in type 2 diabetes patients, the prediction model should adequately reflect the regional differences and accurately predict outcomes in different areas of China. And the raw data did not provide more information on the regional differences. Based on current data, we are unable to build models using populations from some regions and validate the models using populations from other regions. In the future, we can consider designing our studies or collaborating with other researchers to collect as many variables as possible and reduce missing values, including information on regional differences. Therefore, the model we construct could adequately reflect the regional differences and accurately predict outcomes in different areas of China. Fifth, although the performance was tested, it will still need to be tested in clinical or other related settings before it can be widely accepted or applied.

## Conclusion

We have developed and validated a personalized prediction nomogram for the 5-year risk of incident prediabetes in Chinese adults. Our model includes BMI, age, SBP, Scr, FPG, and TG as risk factors. The nomogram demonstrates excellent performance in training and validation cohorts for estimating prediabetes risk and is highly generalizable. Lifestyle, physical activity, and mental health should be considered in further improving the prediabetes risk prediction model. Also, prediabetes risk nomogram still require much clinical and other work before they can be widely adopted and used.

### Supplementary Information

Supplementary Information.

## Data Availability

Data could be downloaded from the ‘DATADRYAD’ database (https://datadryad.org/stash).

## Abbreviations

FPG: Fasting plasma glucose
TC: Total cholesterol
SBP: Systolic blood pressure
BMI: Body mass index
Scr: Serum creatinine
DBP: Diastolic blood pressure
DM: Diabetes mellitus
TG: Triglyceride
LDL-C: Low-density lipid cholesterol
IGT: Impaired glucose tolerance
WC: Waist circumference
ROC: Receiver operating characteristic
ALT: Alanine aminotransferase
ADA: American Diabetes Association
HDL-C: High-density lipoprotein cholesterol
PPV: Positive predictive value
IFG: Impaired fasting glucose
BUN: Blood urea nitrogen
MAR: Missing-at-random
NLR: Negative likelihood ratio
T2DM: Type 2 diabetes mellitus
PLR: Positive likelihood ratio
AIC: Akaike information criterion
LASSO: Least absolute shrinkage and selection operator
NCDRS: New Chinese Diabetes Risk Score
MFP: Multivariable fractional polynomials
NPV: Negative predictive value
CI: Confidence intervals
HR: Hazard ratios
Ref: Reference
AUC: Area under the curve
