# Supplementary material for: A nomogram model for predicting 5-year risk of prediabetes in Chinese adults
Source: Sci Rep. 2023 Dec 18;13:22523. doi: 10.1038/s41598-023-50122-3 (PMC10728122; doi:10.1038/s41598-023-50122-3)
Supplement: Supplementary file 1 — Supplementary Information. [file 41598_2023_50122_MOESM1_ESM.docx]

**A Nomogram Model for Predicting 5-Year Risk of Prediabetes in Chinese Adults**

**Running title: A nomogram for predicting 5-year prediabetes**

**Yanhua Hu^1#^, Yong Han^2,3#^,** **Yufei Liu^4,5#^, Yanan Cui^1^, Zhiping Ni^1^, Ling Wei^1^, Changchun Cao^6*^, Haofei Hu^7,8*^, Yongcheng He^9,10*^,**

^1^College of Information Science and Engineering, Liuzhou Institute of Technology, Liuzhou 545616, Guangxi Zhuang Autonomous Region, China

^2^Department of Emergency, Shenzhen Second People’s Hospital, Shenzhen 518000, Guangdong Province, China

^3^Department of Emergency, The First Affiliated Hospital of Shenzhen University, Shenzhen 518000, Guangdong Province, China

^4^Department of Neurosurgery, Shenzhen Second People’s Hospital, Shenzhen 518000, Guangdong Province, China

^5^Department of Neurosurgery, The First Affiliated Hospital of Shenzhen University, Shenzhen 518000, Guangdong Province, China

^6^Department of Rehabilitation, Shenzhen Dapeng New District Nan’ao People’s Hospital, Shenzhen 518000, Guangdong Province, China

^7^Department of Nephrology, Shenzhen Second People’s Hospital, Shenzhen 518000, Guangdong Province, China

^8^Department of Nephrology, The First Affiliated Hospital of Shenzhen University, Shenzhen 518000, Guangdong Province, China

^9^Department of Nephrology, Shenzhen Hengsheng Hospital, Shenzhen 518000, Guangdong Province, China

^10^Department of Nephrology, Affiliated hospital of North Sichuan Medical College, Nanchong 637000, Sichuan, China

**^#^Yanhua Hu, Yong Han, and Yufei Liu have contributed equally to this work.**

***Corresponding author**

Changchun Cao

Department of Rehabilitation,

Shenzhen Dapeng New District Nan’ao People’s Hospital,

No. 6, Renmin Road, Dapeng New District,

Shenzhen 518000,

Guangdong Province,

China

E-mail: caochangchun1015@163.com

***Corresponding author**

Haofei Hu

Department of Nephrology,

Shenzhen Second People’s Hospital,

No.3002 Sungang Road, Futian District,

Shenzhen 518000,

Guangdong Province,

China

Tel:+86-755-83366388

E-mail: huhaofei0319@126.com

***Corresponding author**

Yongcheng He,

Department of Nephrology,

Shenzhen Hengsheng Hospital,

No. 20 Yintian Road, Baoan District,

Shenzhen 518000,

Guangdong Province,

China

E-mail: heyongcheng640815@126.com

**Table S1 Baseline characteristics of the grouped by missingness of smoking status.**

| Smoking status | Non-missingness group | Missingness group | SD (%) |
| --- | --- | --- | --- |
| Participants | 50979 | 133209 |  |
| Age (year) | 40.29 ± 11.93 | 41.30 ± 12.15 | 8.0% |
| BMI (kg/m^2^) | 23.13 ± 3.27 | 22.94 ± 3.26 | 6.0% |
| SBP (mmHg) | 118.25 ± 15.11 | 117.66 ± 16.06 | 4.0% |
| DBP (mmHg) | 74.07 ± 10.18 | 73.32 ± 10.75 | 7.0% |
| FPG (mmol/L) | 4.79 ± 0.49 | 4.76 ± 0.49 | 7.0% |
| TC(mmol/L) | 4.67 ± 0.88 | 4.67 ± 0.89 | 1.0% |
| TG (mmol/L) | 1.09 (0.76-1.62) | 1.00 (0.70-1.50) | 10.0% |
| HDL-c(mmol/L) | 1.34 ± 0.30 | 1.39 ± 0.31 | 17.0% |
| LDL-c(mmol/L) | 2.72 ± 0.68 | 2.75 ± 0.67 | 5.0% |
| ALT(U/L) | 18.50 (13.00-28.00) | 17.00 (12.30-26.00) | 7.0% |
| AST(U/L) | 22.60 (19.00-27.40) | 21.10 (18.00-26.00) | 11.0% |
| BUN (mmol/L) | 4.60 ± 1.15 | 4.61 ± 1.18 | 1.0% |
| Scr (umol/L) | 71.44 ± 15.09 | 68.97 ± 15.92 | 16.0% |
| Gender |  |  | 29.0% |
| Male | 32251 (63.26%) | 65482 (49.16%) |  |
| Female | 18728 (36.74%) | 67727 (50.84%) |  |
| Incident prediabetes |  |  | 1.0% |
| No | 45141 (88.55%) | 118382 (88.87%) |  |
| Yes | 5838 (11.45%) | 14827 (11.13%) |  |

Values are n(%), mean±SD, or medians (quartiles)

SD, Standardized differences; BMI, Body mass index; AST, Aspartate aminotransferase; SBP, Systolic blood pressure; TC, Total cholesterol; FPG; Fasting plasma glucose; DBP, Diastolic blood pressure; TG, Triglyceride; ALT, Alanine aminotransferase; HDL-c, High-density lipoprotein cholesterol; BUN, Blood urea nitrogen; LDL-c, Low-density lipid cholesterol; Scr, Serum creatinine

**Table S2. Baseline characteristics of the grouped by missingness of drinking status.**

| Drinking status | Non-missingness group | Missingness group | SD (%) |
| --- | --- | --- | --- |
| Participants | 50979 | 133209 |  |
| Age (year) | 40.29 ± 11.93 | 41.30 ± 12.15 | 8.0% |
| BMI (kg/m^2^) | 23.13 ± 3.27 | 22.94 ± 3.26 | 6.0% |
| SBP (mmHg) | 118.25 ± 15.11 | 117.66 ± 16.06 | 4.0% |
| DBP (mmHg) | 74.07 ± 10.18 | 73.32 ± 10.75 | 7.0% |
| FPG (mmol/L) | 4.79 ± 0.49 | 4.76 ± 0.49 | 7.0% |
| TC(mmol/L) | 4.67 ± 0.88 | 4.67 ± 0.89 | 1.0% |
| TG (mmol/L) | 1.09 (0.76-1.62) | 1.00 (0.70-1.50) | 10.0% |
| HDL-c(mmol/L) | 1.34 ± 0.30 | 1.39 ± 0.31 | 17.0% |
| LDL-c(mmol/L) | 2.72 ± 0.68 | 2.75 ± 0.67 | 5.0% |
| ALT(U/L) | 18.50 (13.00-28.00) | 17.00 (12.30-26.00) | 7.0% |
| AST(U/L) | 22.60 (19.00-27.40) | 21.10 (18.00-26.00) | 11.0% |
| BUN (mmol/L) | 4.60 ± 1.15 | 4.61 ± 1.18 | 1.0% |
| Scr (umol/L) | 71.44 ± 15.09 | 68.97 ± 15.92 | 16.0% |
| Gender |  |  | 29.0% |
| Male | 32251 (63.26%) | 65482 (49.16%) |  |
| Female | 18728 (36.74%) | 67727 (50.84%) |  |
| Incident prediabetes |  |  | 1.0% |
| No | 45141 (88.55% | 118382 (88.87%) |  |
| Yes | 5838 (11.45%) | 14827 (11.13%) |  |

Values are n(%), mean±SD, or medians (quartiles)

SD, Standardized differences; BMI, Body mass index; AST, Aspartate aminotransferase; SBP, Systolic blood pressure; TC, Total cholesterol; FPG; Fasting plasma glucose; DBP, Diastolic blood pressure; TG, Triglyceride; ALT, Alanine aminotransferase; HDL-c, High-density lipoprotein cholesterol; BUN, Blood urea nitrogen; LDL-c, Low-density lipid cholesterol; Scr, Serum creatinine

**Table S3. Baseline characteristics of the grouped by missingness of HDL-c.**

| HDL-c | Non-missingness group | Missingness group | SD (%) |
| --- | --- | --- | --- |
| Participants | 100837 | 83351 |  |
| Age (year) | 42.89 ± 12.45 | 38.75 ± 11.24 | 35.0% |
| BMI (kg/m^2^) | 23.09 ± 3.22 | 22.87 ± 3.31 | 7.0% |
| SBP (mmHg) | 118.05 ± 16.07 | 117.55 ± 15.47 | 3.0% |
| DBP (mmHg) | 73.74 ± 10.76 | 73.27 ± 10.39 | 4.0% |
| FPG (mmol/L) | 4.78 ± 0.48 | 4.75 ± 0.50 | 8.0% |
| TC(mmol/L) | 4.75 ± 0.88 | 4.57 ± 0.88 | 2.0% |
| TG (mmol/L) | 1.06 (0.73-1.58) | 1.00 (0.70-1.48) | 8.0% |
| LDL-c(mmol/L) | 2.74 ± 0.67 | 2.79 ± 0.61 | 7.0% |
| ALT(U/L) | 17.70 (12.70-26.50) | 17.20 (12.30-26.60) | 1.0% |
| AST(U/L) | 21.90 (18.30-26.00) | 21.50 (18.20-26.00) | 0.0% |
| BUN (mmol/L) | 4.63 ± 1.16 | 4.58 ± 1.18 | 5.0% |
| Scr (umol/L) | 69.90 ± 15.72 | 69.34 ± 15.74 | 4.0% |
| Gender |  |  | 5.0% |
| Male | 52388 (51.95%) | 45345 (54.40%) |  |
| Female | 48449 (48.05%) | 38006 (45.60%) |  |
| Incident prediabetes |  |  | 7.0% |
| No | 88474 (87.74%) | 75049 (90.04%) |  |
| Yes | 12363 (12.26%) | 8302 (9.96%) |  |

Values are n(%), mean±SD, or medians (quartiles)

SD, Standardized differences; BMI, Body mass index; AST, Aspartate aminotransferase; SBP, Systolic blood pressure; TC, Total cholesterol; FPG; Fasting plasma glucose; DBP, Diastolic blood pressure; TG, Triglyceride; ALT, Alanine aminotransferase; HDL-c, High-density lipoprotein cholesterol; BUN, Blood urea nitrogen; LDL-c, Low-density lipid cholesterol; Scr, Serum creatinine

**Table S4. Baseline characteristics of the grouped by missingness of AST.**

| AST | Non-missingness group | Missingness group | SD (%) |
| --- | --- | --- | --- |
| Participants | 76533 | 107655 |  |
| Age (year) | 40.89 ± 12.12 | 41.11 ± 12.08 | 2.0% |
| BMI (kg/m^2^) | 23.01 ± 3.25 | 22.97 ± 3.27 | 1.0% |
| SBP (mmHg) | 118.02 ± 15.91 | 117.68 ± 15.74 | 2.0% |
| DBP (mmHg) | 73.48 ± 10.63 | 73.56 ± 10.58 | 1.0% |
| FPG (mmol/L) | 4.78 ± 0.49 | 4.76 ± 0.48 | 4.0% |
| TC(mmol/L) | 4.65 ± 0.89 | 4.68 ± 0.88 | 3.0% |
| TG (mmol/L) | 1.00 (0.70-1.52) | 1.03 (0.72-1.54) | 2.0% |
| HDL-c(mmol/L) | 1.38 ± 0.30 | 1.38 ± 0.32 | 2.0% |
| LDL-c(mmol/L) | 2.74 ± 0.66 | 2.74 ± 0.68 | 0.0% |
| ALT(U/L) | 17.80 (12.70-27.00) | 17.30 (12.50-26.30) | 2.0% |
| BUN (mmol/L) | 4.64 ± 1.19 | 4.59 ± 1.15 | 4.0% |
| Scr (umol/L) | 70.84 ± 15.93 | 68.80 ± 15.53 | 13.0% |
| Gender |  |  | 8.0% |
| Male | 42383 (55.38%) | 55350 (51.41%) |  |
| Female | 34150 (44.62%) | 52305 (48.59%) |  |
| Incident prediabetes |  |  | 9.0% |
| No | 66623 (87.05%) | 96900 (90.01%) |  |
| Yes | 9910 (12.95%) | 10755 (9.99%) |  |

Values are n(%), mean±SD, or medians (quartiles)

SD, Standardized differences; BMI, Body mass index; AST, Aspartate aminotransferase; SBP, Systolic blood pressure; TC, Total cholesterol; FPG; Fasting plasma glucose; DBP, Diastolic blood pressure; TG, Triglyceride; ALT, Alanine aminotransferase; HDL-c, High-density lipoprotein cholesterol; BUN, Blood urea nitrogen; LDL-c, Low-density lipid cholesterol; Scr, Serum creatinine

**Table S5. Risk predictors for incident prediabetes in the univariate and multivariate analysis.**

| Exposure | Univariable (HR,95%CI, P) | Multivariable (HR,95%CI, P) |
| --- | --- | --- |
| Age(year) | 1.034 (1.032, 1.035) <0.00001 | 1.020 (1.019, 1.022) <0.00001 |
| Gender |  |  |
| Male | Ref. | Ref. |
| Female | 0.622 (0.597, 0.647) <0.00001 | 0.918 (0.863, 0.977) 0.00700 |
| BMI (kg/m^2^) | 1.121 (1.115, 1.127) <0.00001 | 1.047 (1.040, 1.055) <0.00001 |
| SBP (mmHg) | 1.025 (1.024, 1.026) <0.00001 | 1.008 (1.007, 1.010) <0.00001 |
| DBP (mmHg) | 1.029 (1.027, 1.031) <0.00001 | 1.003 (1.000, 1.005) 0.02454 |
| FPG (mmol/L) | 5.728 (5.451, 6.020) <0.00001 | 4.611 (4.386, 4.848) <0.00001 |
| TG (mmol/L) | 1.186 (1.175, 1.197) <0.00001 | 1.067 (1.050, 1.085) <0.00001 |
| HDL-c (mmol/L) | 0.733 (0.689, 0.781) <0.00001 | 1.214 (1.133, 1.301) <0.00001 |
| LDL-c (mmol/L) | 1.244 (1.211, 1.278) <0.00001 | 0.947 (0.920, 0.976) 0.00030 |
| ALT (U/L) | 1.004 (1.003, 1.004) <0.00001 | 1.003 (1.002, 1.005) 0.00003 |
| AST (U/L) | 1.006 (1.005, 1.007) <0.00001 | 0.998 (0.995, 1.001) 0.15685 |
| BUN (mmol/L) | 1.145 (1.127, 1.163) <0.00001 | 0.967 (0.950, 0.984) 0.00015 |
| Scr (umol/L) | 1.015 (1.013, 1.016) <0.00001 | 1.006 (1.004, 1.007) <0.00001 |
| Smoking status |  |  |
| Current | Ref. | Ref. |
| Ever | 0.894 (0.808, 0.990) 0.03098 | 0.974 (0.879, 1.079) 0.61262 |
| Never | 0.700 (0.668, 0.734) <0.00001 | 0.991 (0.940, 1.044) 0.73114 |
| Drinking status |  |  |
| Current | Ref. | Ref. |
| Ever | 0.784 (0.688, 0.894) 0.00026 | 1.022 (0.895, 1.167) 0.74662 |
| Never | 0.599 (0.530, 0.677) <0.00001 | 1.053 (0.928, 1.195) 0.42052 |
| Family history |  |  |
| No | Ref. | Ref. |
| Yes | 0.981 (0.862, 1.117) 0.77269 | 1.080 (0.949, 1.231) 0.24399 |

BMI, Body mass index; SBP, Systolic blood pressure; DBP, Diastolic blood pressure; FPG; Fasting plasma glucose; TG, Triglyceride; HDL-C, High-density lipoprotein cholesterol; LDL-C, Low-density lipid cholesterol; ALT, Alanine aminotransferase; BUN, Blood urea nitrogen; Scr, Serum creatinine; AST, Aspartate aminotransferase.

HR, Hazard ratios; CI, Confidence interval; Ref, Reference.

**Table S6. Prediction performance of risk predictors in our nomogram.**

|  | Age | Training  BMI | cohort  SBP | FPG | TG | Scr | Age | Validation  BMI | cohort  SBP | FPG | TG | Scr |
| --- | --- | --- | --- | --- | --- | --- | --- | --- | --- | --- | --- | --- |
| AUC | 0.6455 | 0.6333 | 0.6290 | 0.6822 | 0.6116 | 0.5679 | 0.6422 | 0.6341 | 0.6293 | 0.6836 | 0.6161 | 0.5658 |
| 95% CI |  |  |  |  |  |  |  |  |  |  |  |  |
| Lower | 0.6398 | 0.6278 | 0.6233 | 0.6767 | 0.6059 | 0.5621 | 0.6365 | 0.6286 | 0.6236 | 0.6781 | 0.6103 | 0.5600 |
| Upper | 0.6512 | 0.6388 | 0.6347 | 0.6878 | 0.6174 | 0.5737 | 0.6479 | 0.6396 | 0.6350 | 0.6891 | 0.6218 | 0.5715 |
| Best threshold | 42.0 | 22.69 | 117.50 | 5.02 | 1.10 | 66.98 | 42.0 | 22.75 | 120.50 | 4.93 | 1.09 | 64.86 |
| Specificity, % | 68.39 | 53.69 | 54.89 | 69.70 | 61.86 | 47.29 | 63.82 | 52.58 | 61.93 | 62.18 | 61.73 | 42.79 |
| Sensitivity, % | 53.80 | 66.38 | 63.34 | 57.68 | 55.54 | 63.60 | 57.67 | 67.48 | 56.71 | 65.45 | 56.13 | 67.55 |
| Accuracy, % | 66.75 | 55.11 | 55.84 | 68.35 | 61.15 | 49.12 | 63.13 | 54.25 | 61.35 | 62.54 | 61.10 | 45.56 |
| PPV, % | 17.72 | 15.35 | 15.08 | 19.41 | 15.56 | 13.24 | 16.75 | 15.23 | 15.83 | 17.93 | 15.62 | 12.79 |
| NPV, % | 92.13 | 92.66 | 92.21 | 92.87 | 91.67 | 91.13 | 92.27 | 92.76 | 91.89 | 93.45 | 91.77 | 91.26 |
| PLR | 1.7020 | 1.4333 | 1.4041 | 1.9033 | 1.4562 | 1.2067 | 1.5940 | 1.4230 | 1.4898 | 1.7304 | 1.4666 | 1.1806 |
| NLR | 0.6756 | 0.6262 | 0.6679 | 0.6072 | 0.7187 | 0.7697 | 0.6633 | 0.6185 | 0.6989 | 0.5557 | 0.7107 | 0.7585 |
| DOR | 2.5194 | 2.2891 | 2.1022 | 3.1345 | 2.0262 | 1.5677 | 2.4031 | 2.3005 | 2.1315 | 3.1141 | 2.0637 | 1.5565 |
| P-value | <0.001 | <0.001 | <0.001 | Ref. | <0.001 | <0.001 | <0.001 | <0.001 | <0.001 | Ref. | <0.001 | <0.001 |

AUC, Area under curve; CI, Confidence interval; PPV, Positive predictive value; NPV, Negative predictive value; PLR, Positive likelihood ratio; NLR, Negative likelihood ratio; DOR, Diagnostic odds ratio.

P-value: Compared with FPG

**Table S7. Prediction performance of** **the nomogram for the risk of prediabetes** **in participants with BMI≥ 24kg/m^2^.**

| AUC | 95%  Lower | CI  Upper | Best threshold of  predicted prediabetes probability | Specificity  (%) | Sensitivity  (%) | PPV  (%) | NPV  (%) | PLR | NLR |
| --- | --- | --- | --- | --- | --- | --- | --- | --- | --- |
| 0.6963 | 0.6908 | 0.7018 | 0.4743 | 65.50 | 63.58 | 25.41 | 90.68 | 1.843 | 0.556 |

AUC, Area under the curve; CI, Confidence interval; NPV, Negative predictive value; PPV, Positive predictive value; NLR, Negative likelihood ratio; PLR, Positive likelihood ratio;

**Figure S1. The ROC curves of each risk predictor in our stepwise model, including age, BMI, SBP, FPG, Scr and TG in the training cohort (A) and validation cohort (B).**


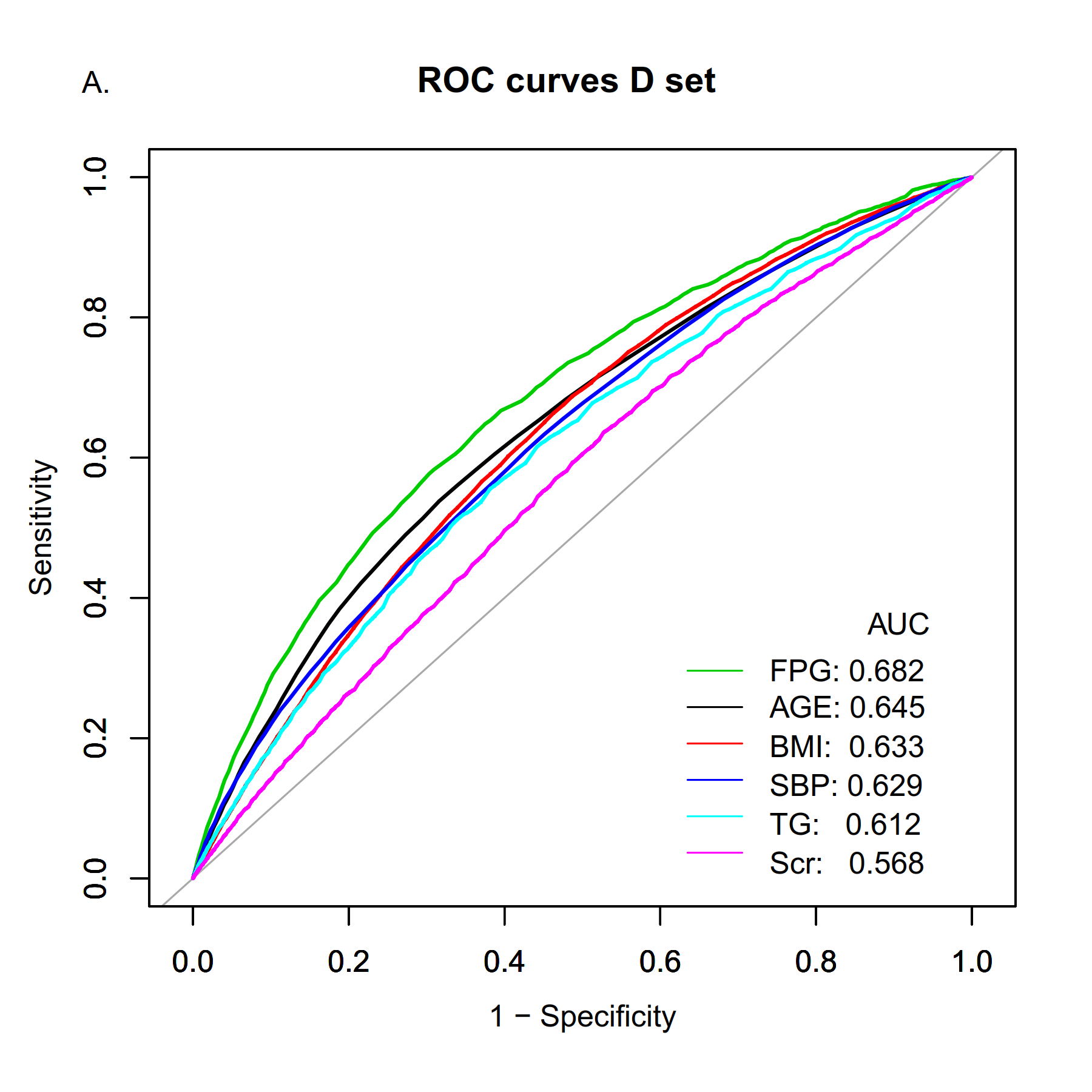


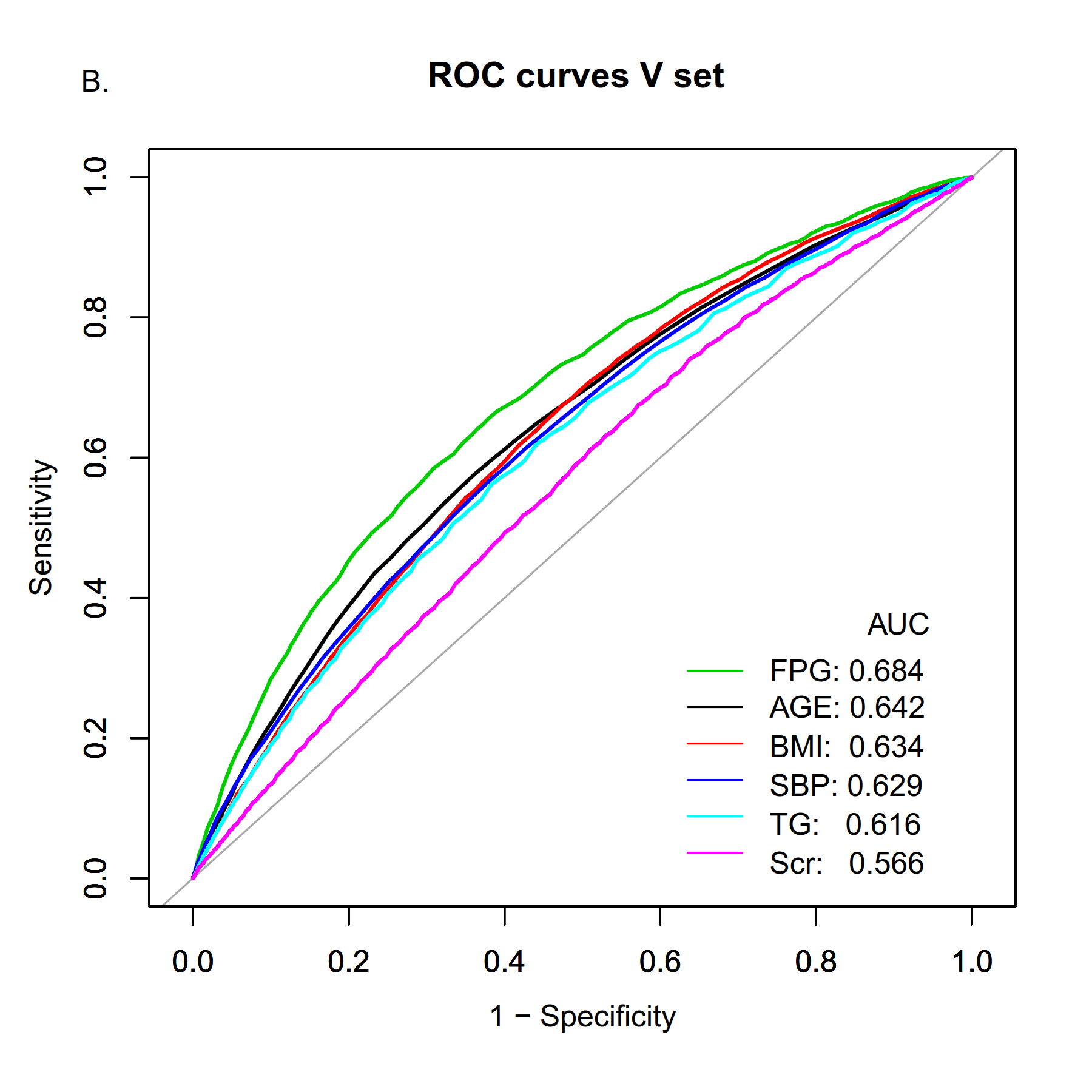


Figure S1. The AUC of the FPG was greater than the AUC of other risk factors for the 5-year incident prediabetes.

**Figure S2. Comparing the predicted prediabetes probability between the prediabetes and non-prediabetes groups in the training cohort (A) and validation cohort (B).**


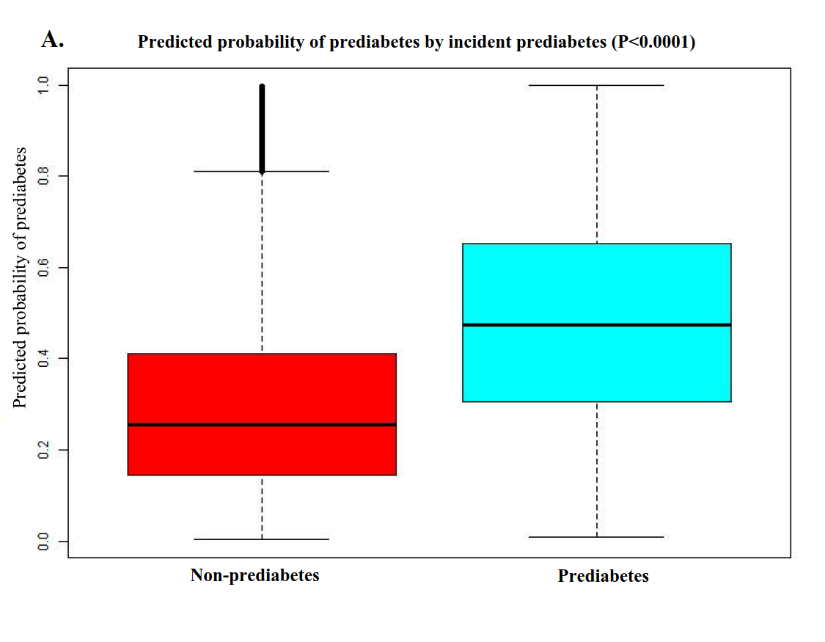


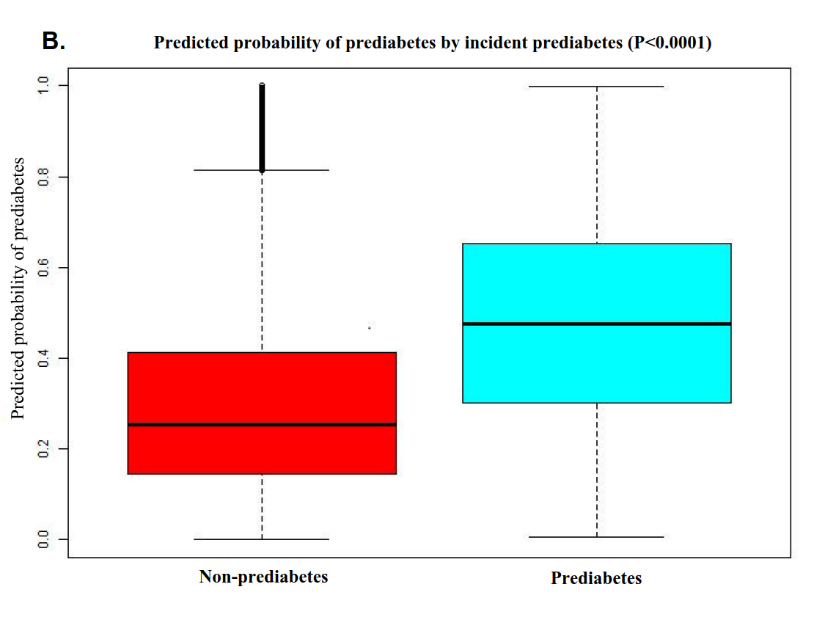


Figure S2 showed that participants with prediabetes had a higher predicted probability, whereas those without prediabetes had a lower predicted probability.

**Figure S3. ROC curve for the nomogram predicting the risk of developing prediabetes at 5 years in the participants with BMI≥24kg/m^2^.**


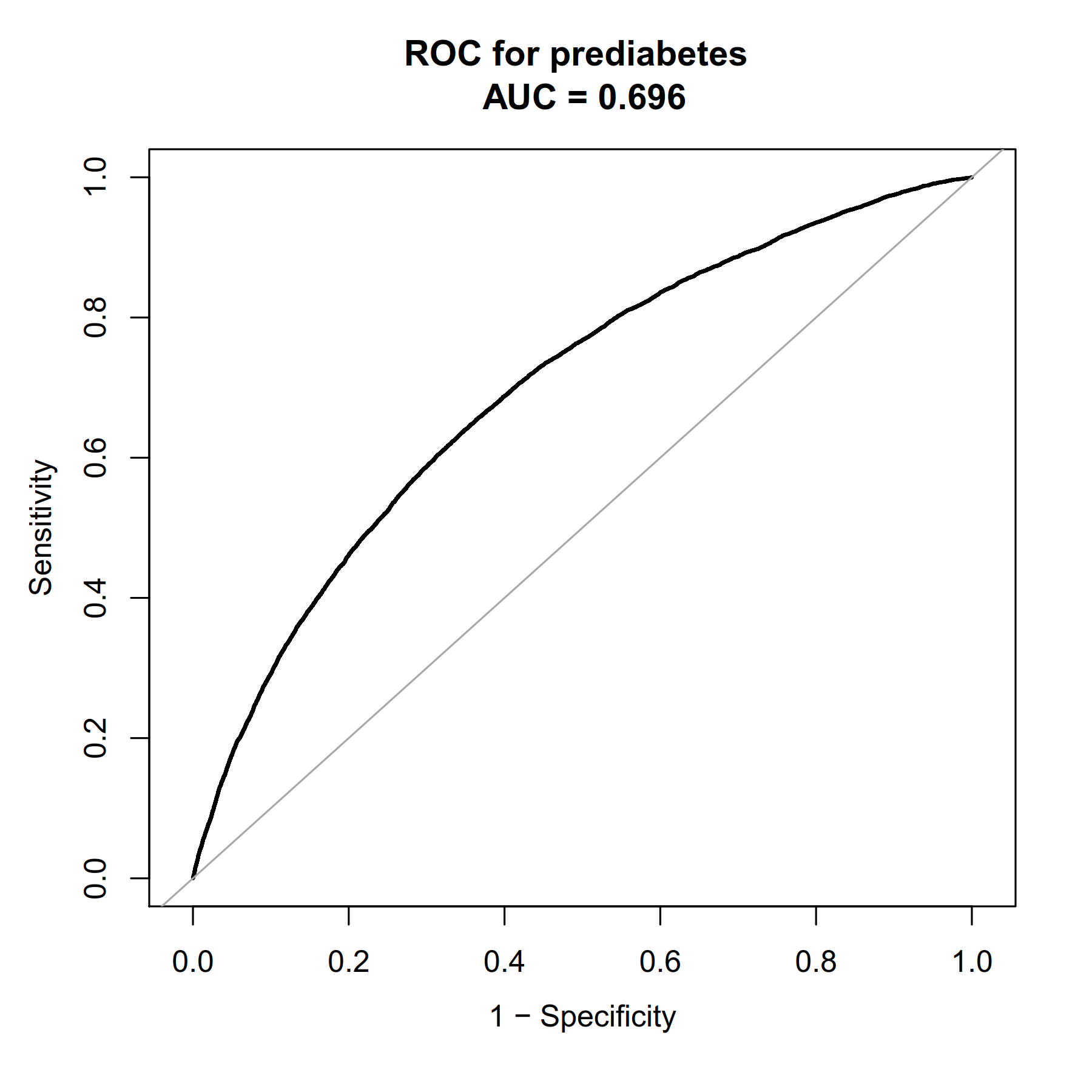


Figure S3. In the participants with BMI≥24kg/m^2^, AUC of the nomogram were 0.696.

**Figure S4. The decision curve analysis of the nomogram model for 5-year prediabetes risk in the participants with BMI≥24kg/m^2^.**


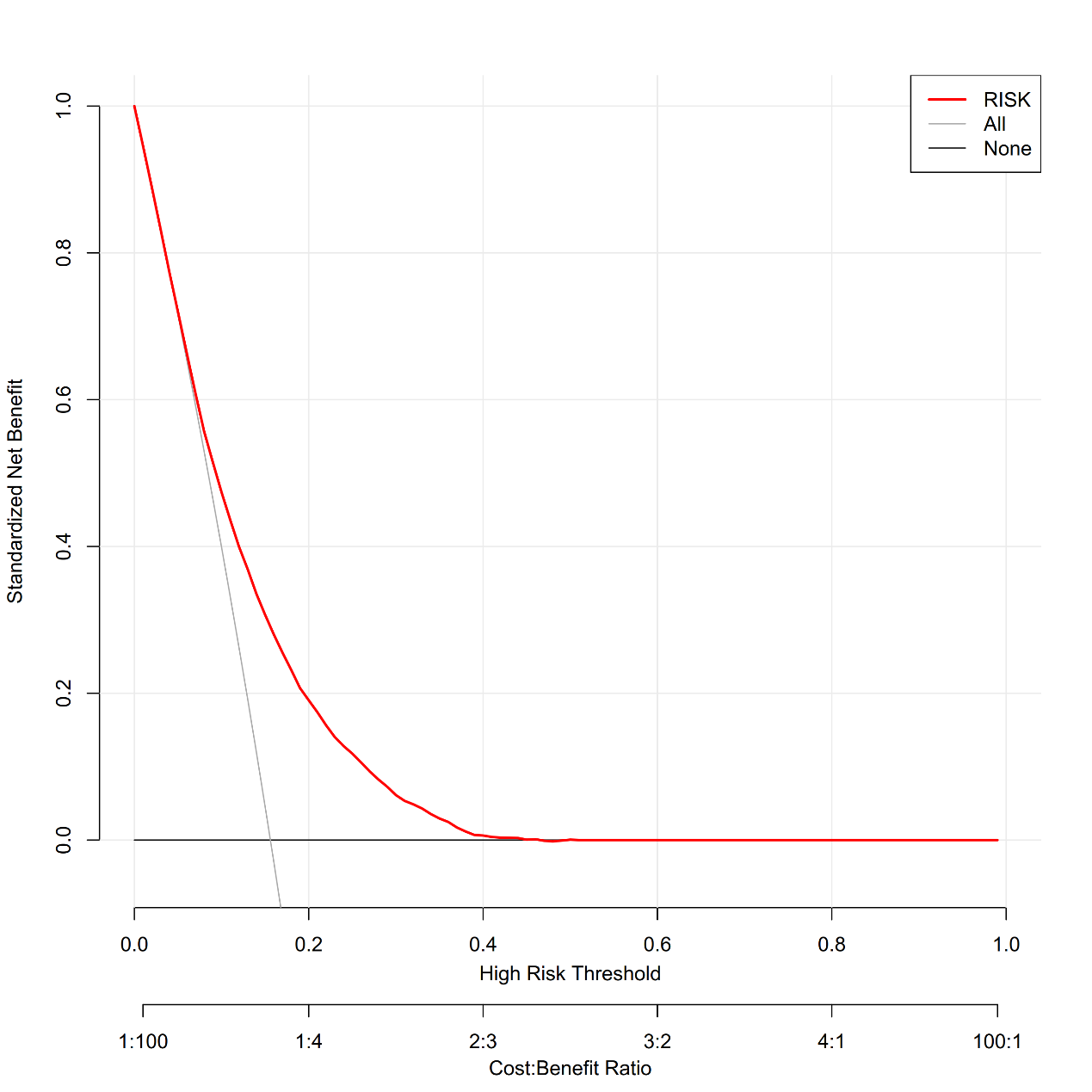


Figure S4. When no participant is thought to acquire prediabetes, the black line shows the net benefit. When prediabetes is considered for all participants, the light gray line represents the net benefit. A model's clinical utility is indicated by the area between the "no treatment line" (black line) and the "all treatment line" (light gray line). The more distance between the model curve and the black and light gray lines, the better the nomogram's clinical value.
